# Effect of surgical antimicrobial prophylaxis duration for colic surgery on complications and resistome

**DOI:** 10.1002/evj.70137

**Published:** 2025-12-10

**Authors:** Louise L. Southwood, Alicia Long, Jairo Perez, Scott Daniel, Kyle Bittinger, Maia Aitken, Laurel Redding

**Affiliations:** ^1^ Department of Clinical Studies, New Bolton Center University of Pennsylvania Kennett Pennsylvania USA; ^2^ Division of Gastroenterology, Hepatology and Nutrition Children's Hospital of Philadelphia Philadelphia Pennsylvania USA

**Keywords:** colic, complication, gastrointestinal, horse, infection, laparotomy

## Abstract

**Background:**

Based on human studies, surgical antimicrobial (AMD) prophylaxis (SAP) beyond 24 h is unnecessary and potentially detrimental.

**Objective:**

To compare clinical and microbiological outcomes in patients receiving 24‐ or 72‐h of SAP for colic surgery.

**Study Design:**

Prospective randomised clinical trial.

**Methods:**

Horses that recovered from colic surgery were considered. Exclusion criteria were (1) age <2 years; (2) Miniature Horses, pony, and draught breeds; (3) azotaemia; (4) recent hospitalisation, colic surgery, or AMDs; (5) local AMD administration. Eligible horses were randomly assigned to receive SAP with potassium penicillin and gentamicin for 24‐ or 72‐h. Clinical data and complications were compared between SAP groups. Admission and discharge faecal samples from a subset of horses (*N* = 49) underwent shotgun metagenomic sequencing on an Illumina platform. Host reads were filtered by aligning to reference genomes using the Burrows–Wheeler Aligner, and taxonomic classification was performed with kraken2. Sequencing reads were aligned to the Comprehensive Antimicrobial Resistance Database (CARD)5 and characterised using the AMR++ pipeline. The microbiome/resistome was characterised and compared between SAP groups over time.

**Results:**

One hundred and forty horses completed the study (24‐h *N* = 71 and 72‐h *N* = 69). The only clinical variable that was different between SAP groups was age (24‐h median age 16 [IQR 9, 20] and 72‐h 12 [6, 18] years, *p* = 0.03). There was no significant difference between groups for any complications including incisional infection (24‐h 17 [95% CI 10–27]% and 72‐h 16 [9–26]%, *p* = 0.9). Time was the main driver of changes in the microbiome/resistome: alpha diversity decreased while AMD resistance genes associated with administered AMD increased between admission and discharge. Discharge beta‐lactam resistance genes were significantly higher in the 72‐h than the 24‐h group.

**Main Limitations:**

Single hospital, small numbers for complications, clinicians not blinded to SAP group.

**Conclusions:**

SAP for 24‐h is recommended for horses undergoing colic surgery.

## 1 INTRODUCTION

Antimicrobial resistance (AMR) in horses is an understudied and increasingly concerning problem. Numerous antimicrobial‐resistant pathogens have been reported in horses, including extended‐spectrum beta‐lactamase (ESBL)‐producing *Escherichia coli*, methicillin‐resistant *Staphylococcus aureus* (MRSA), and multidrug‐resistant (MDR) *Salmonella* spp.^1^, ^2^, ^3^, ^4^ Infections with these pathogens can result in increased morbidity, mortality, and treatment costs in horses, as well as the potential zoonotic transfer of pathogens to horse owners and personnel in contact with horses. Antimicrobial drugs (AMD) are frequently used in equine medicine,^5^, ^6^, ^7^ including critically important AMD such as fluoroquinolones and third/fourth generation cephalosporins.^8^ Because any use of AMD can result in AMR, and because AMD can have adverse effects on the equine gastrointestinal (GI) tract and microbiome,^9^, ^10^ there is an urgent need to determine when AMD use in horses can safely be decreased or even eliminated. Veterinarians consistently report being very motivated to find ways of decreasing AMD use.^11^, ^12^, ^13^, ^14^, ^15^ Unfortunately, because of a dearth of evidence‐based guidelines on AMD use in veterinary medicine—especially related to the duration of surgical AMD prophylaxis (SAP)—it is difficult for clinicians to know whether and when they can safely reduce the duration of their patients' AMD regimens.

Antimicrobials are often used perioperatively in horses undergoing surgery. However, unlike in human medicine, where expansive guidelines on SAP exist,^16^ there are no evidence‐based guidelines for SAP in equine surgery. Because colic surgeries are often clean‐contaminated procedures or prolonged in duration, SAP is usually indicated. However, there is no consensus on the required duration of SAP. In human medicine, short durations (<24 h) of SAP for abdominal surgeries are recommended.^16^ Antimicrobial regimens in equine medicine typically exceed 24 h,^17^ perhaps due to the postoperative (PO) environments in which horses are kept, where infection control is more challenging^18^ and where multidrug resistant infections tend to dominate.^18^, ^19^ While shorter durations of SAP are preferred to minimise the selection pressure for AMR and the likelihood of adverse effects of AMDs on the equine hindgut, they must be weighed against the possibility of surgical site infections (SSI) and post‐anaesthetic pneumonia.

Several small‐scale clinical trials comparing various durations of SAP in horses undergoing colic surgery have been performed, including a comparison of 3 days vs. 5 days,^17^ one dose vs. 5 days,^20^ pre‐ and intra‐operative vs. 3 days,^21^ and retrospective studies evaluating different durations of SAP.^22^, ^23^, ^24^ None of these studies have found a significant difference in any of the clinical outcomes between the groups, including SSI, PO colitis, and other PO complications, except for the study comparing pre‐ and intra‐operative vs. 3 days of AMD where horses receiving 3 days of AMD had a lower rate of SSI.^21^ Clinical outcomes that directly and immediately affect the health of the patient—such as SSIs—are crucial for patient care, and any study evaluating different durations of SAP should verify that shorter durations of AMD therapy are not associated with worse clinical outcomes. However, microbiological outcomes, particularly those related to AMR, are also important determinants of both the short‐ and long‐term health of the individual patient and the larger equine ‘herd’. No studies have assessed the effect of different durations of SAP regimens on microbial outcomes in horses undergoing colic surgery. It is suspected that longer durations will be associated with a greater likelihood of isolating AMD‐resistant bacteria from the patient, as has been found in humans undergoing surgery,^25^, ^26^ as well as an expansion of the resistome (collection of AMR genes in the GI tract). Our hypotheses, therefore, were that shorter durations of SAP for colic surgery (1) produce similar rates of PO complications and (2) less AMR than longer durations. The primary objective of this study to address our hypotheses was thus to compare clinical and microbiological outcomes in patients receiving 24‐h or 72‐h of SAP for colic surgery and to better understand how the equine faecal microbiome and resistome change over the course of hospitalisation and recovery from surgery and SAP. Clinical variables associated with each PO complication were also explored as a secondary objective.

## 2 MATERIALS AND METHODS

### 2.1 Horses and clinical data

Horses admitted to a tertiary care facility between 8 June 2022 and 13 January 2025 for colic caused by GI disease that underwent exploratory laparotomy were considered for study inclusion. Only horses that recovered from surgery and general anaesthesia were considered. Exclusion criteria were (1) age <2 years old; (2) Miniature Horses, and pony and draught breeds; (3) azotaemia (creatinine concentration >88.42 μmol/L); (4) hospitalisation, colic surgery, or AMD therapy in the previous 3 months; and (5) intraperitoneal or peri‐incisional AMD administration.

Horses were randomised via block‐randomisation to receive either 24‐h or 72‐h of SAP. A piece of paper labelled with a 24‐ or 72‐h group assignment was placed into an envelope in blocks of 20 (i.e., 10 per group in each block) and the envelopes were consecutively numbered and placed in numerical order. With a target enrolment of 150 horses and 1:1 allocation to each treatment group, our study was powered to detect a one‐sided difference in rates of SSI of 15% given the previously observed rate of 18% in our hospital.^27^ A difference of 15% was selected to identify a clinically relevant distinction as has been observed in another study^21^ and 150 horses was considered a reasonable number to recruit with the initially proposed study period of 2 years. At the completion of surgery, the next numerically ordered envelope containing the 24‐h or 72‐h SAP group assignment was selected by the operating room nurse and given to the house officer assigned to the patient. Horses that were euthanased within 12 days of surgery for reasons that were clearly unrelated to infection (e.g., colic and shock caused by bowel necrosis) were excluded and the reason for exclusion justified and clearly documented (see Results section). Horses that developed an infection within the 12‐day period were included. Note that the durations for SAP were based on current use in our hospital versus the duration that is used by other surgeons based on discussions at continuing education meetings.

Information obtained on each horse included signalment (age, breed, sex), bodyweight, and colic duration. Recorded physical examination findings included rectal temperature, heart rate, respiratory rate, mucus membrane colour/moistness, and capillary refill time. Other admission variables included volume of reflux on nasogastric intubation and laboratory values (packed cell volume, total solids, blood lactate, glucose, creatinine, and electrolyte concentrations), and, where relevant, the colour, TS, and lactate concentration of peritoneal fluid.

Horses were administered potassium penicillin (22,000 units/kg intravenously [IV]) and gentamicin (8.8 mg/kg IV) within an hour of starting surgery, defined as the time of first incision. A second dose of potassium penicillin was given if the procedure extended beyond 2 h. Timing of AMD administration relative to starting surgery, anaesthesia and surgery duration, number of times potassium penicillin was re‐dosed during surgery, lowest mean arterial pressure (MAP), lowest oxygen (PaO_2_), highest carbon dioxide (PaCO_2_) partial pressures, PCV, TS, and lactate concentration during surgery were recorded. The type of wound protection (iodine‐impregnated adhesive drape (Ioban®), stent with an Ioban® or stent only) and whether it became dislodged during recovery, as well as the duration of recovery, were recorded. The most senior surgeon performing the procedure was identified and surgeons were categorised as: Surgeon (ACVS Diplomate for at least 6 years), Junior Surgeon (ACVS or ACVECC Diplomate for fewer than 6 years), or Resident (ACVS third‐year surgery resident).

Postoperative variables included placement and duration of abdominal bandage, duration of flunixin meglumine, administration of IV lidocaine, IV fluid rate and duration of IV fluids as well as any additives (KCl, calcium gluconate, or dextrose), and PO feeding (time to first feed, type of first feed, time to full feed and type of full feed). Horses in the 24‐h SAP group received an additional three doses of potassium penicillin (22,000 units/kg IV q6h) and horses in the 72‐h group received 2 additional doses of gentamicin (8.8 mg/kg IV q24h) and 11 additional doses of potassium penicillin.

### 2.2 Postoperative complications

Data collected on PO infection‐related complications included occurrence, timing, severity (slight 38.3–38.8°C, mild 38.9–39.4°C, moderate 39.5–39.9°C, marked ≥40°C), and duration of PO pyrexia; SSI, defined as persistent drainage for >36 h of serous, purulent, or serosanguineous fluid from the incision that occurs after the initial 48‐h PO period and was treated either locally or systemically; IV catheter‐associated infection, defined as heat pain, swelling, and/or drainage at the IV catheter site; pneumonia, defined by signs of fever, tachypnea, nasal discharge, coughing, and supported by radiographic or sonographic findings; and septic peritonitis, defined as fever or dull demeanour supported by sonographic or relaparotomy findings and peritoneal fluid analysis. If pneumonia or septic peritonitis occurred for an obvious reason, such as necrotic bowel or leakage from an enterotomy/enterectomy site, or severe aspiration of gastric contents during surgery, the horse was excluded, and the reason for exclusion justified and documented (see Results section).

Data on PO GI‐related complications included: occurrence, duration, and severity of colic; need for a relaparotomy and diagnosis at relaparotomy; occurrence of inappetence/anorexia defined as reduced or no interest in any type of feed; occurrence, volume, and timing of PO nasogastric reflux defined as >20 L over 24 h or >8 L at any single sampling time; and diarrhoea defined as >2 episodes of loose faeces in any 24‐h period. Finally, the length of stay and cost of treatment were recorded. Follow‐up was a minimum of 1 month after surgery by unscripted telephone conversation, text, or email with the owner/caregiver or primary care veterinarian.

### 2.3 Sample collection

Admission faecal samples were collected from all horses admitted for colic during their routine initial physical examination. If no faecal material was available for collection from the rectum, the rectal mucosa was swabbed. A faecal sample was collected from the stall floor of all participating horses at the time of hospital discharge. It has been shown that the microbiota at the centre of faecal balls on the stall floor are representative of the faecal microbiota obtained directly per rectum,^28^ though we acknowledge that subtle changes could occur depending on how long the samples are exposed to air. All faecal samples were stored at −80°C and processed in batch.

### 2.4 Shotgun metagenomic sequencing

Admission and discharge samples from a subset of 25 horses in the 24‐h group, 19 horses in the 72‐h group, and 5 medically managed horses (receiving no AMDs) as temporal controls were selected for shotgun metagenomic sequencing. With this number, the study was adequately powered to detect differences of *d* = 0.6 standard deviations (SD) in the abundance of the most abundant AMR genes. Shotgun libraries were generated from 1 ng of DNA using the NexteraXT kit (Illumina) and sequenced on the Illumina NextSeq using 2 × 150‐base pair (bp) chemistry. To assess environmental and reagent contamination, extraction blanks and DNA‐free water were processed in parallel with experimental samples. Paired‐end reads were filtered by aligning to reference genomes using the Burrows–Wheeler Aligner (BWA),^29^ and taxonomic classification was performed with kraken2.^30^ Sequencing reads were also aligned to the Comprehensive Antimicrobial Resistance Database (CARD)^5^ 5 and characterised using the AMR++ pipeline, which classifies AMR genes by gene, group, mechanism of action, and antibiotic class. To normalise the abundance of AMR genes in each sample, Antibiotic Resistance Ontology (ARO) counts were divided by the total number of nonhost reads (in millions) and the ARO length (in kilobases) to generate Reads per Kilobase per Million (RPKM) reads. Zero counts were replaced by 1/2 the minimum value for any given ARO. Finally, a logarithm of base 2 was taken for the relative abundance to account for skewed distributions. Taxonomic abundances were treated similarly except they were normalised to the total count of mapped reads in each sample and were not adjusted for genome lengths of the given species.

### 2.5 Data analysis—Clinical outcomes

Univariable analysis was performed using Fisher's exact or chi‐squared test, Student's *t* or Wilcoxon rank sum test as appropriate to determine the unadjusted association between SAP group and signalment, admission clinical, surgical, and anaesthesia data, and each PO complication. Univariable analysis was also performed to determine clinical variables associated with each PO complication. Variables with an association for which *p* < 0.1 were considered for multivariable analysis, which was performed using a backward stepwise regression (linear or logistic, as appropriate). Only variables with *p* < 0.05 were included in the final models. Data retained in the final multivariable model are compared using odds ratios (OR) and 95% confidence intervals (CI). All data analyses were performed using JMP® Pro 17 Statistical Analysis System. Based on these analyses, it was determined if (1) the randomisation for SAP group assignment was adequate, (2) there was a significant difference between SAP group and PO complications, and (3) variables associated with each PO complication. Categorical data are reported as numbers and percentages with 95% confidence intervals for the main comparisons and continuous data as median and interquartile range (IQR) presented as the 25th and 75th percentiles.

### 2.6 Data analysis—Microbial outcomes

Primary metagenomic outcomes included the difference in alpha diversity and composition of the faecal microbiome and resistome between admission (i.e., prior to AMD therapy) and discharge and between SAP groups using permutative analysis of variance (PERMANOVA). The differential abundance of the most frequently encountered AMR genes and gene families between SAP groups and across time points was also examined. Linear mixed‐effect models were used to estimate changes in log2‐transformed RPKM between admission and discharge and between study groups at discharge. Multiple tests were adjusted for false discovery rate using the Benjamini‐Hochberg method. All analyses were conducted with R Studio, two‐sided tests of hypotheses, and a p‐value <0.05 as the criterion for statistical significance. To do a sensitivity analysis, we employed two approaches: (1) adding pseudocounts and (2) zero‐inflated beta random effect model. Pseudocounts of 1 were added to both the numerator and denominator during the count normalisation. The zero‐inflated beta random effect model (ZIBR) uses a split‐approach by using both logistic models to account for sparse genes (i.e., those with >50% zeros) and a beta regression model to account for more abundant genes with few zeros.^31^

## 3 RESULTS

### 3.1 Horses and clinical data

**FIGURE 1:**
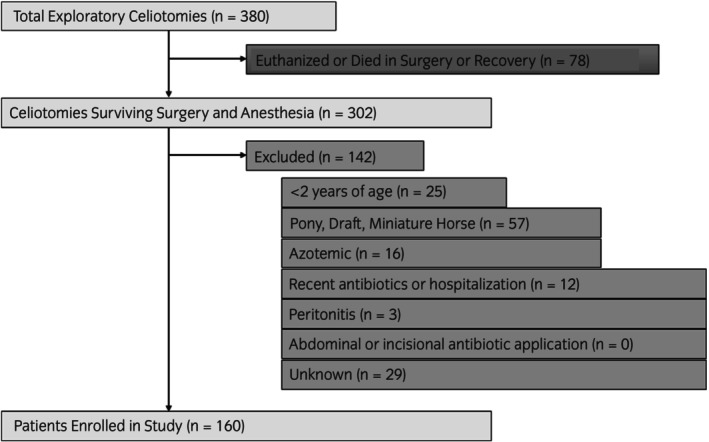
Flow chart showing case inclusion and exclusion.

Three hundred and eighty horses underwent exploratory laparotomy during the study period, with 302 having recovered from surgery and general anaesthesia. One hundred and forty‐two horses were excluded for age <2 years old (*n* = 25), Miniature Horse, pony, or draught breed (*n* = 57), azotemia (*n* = 16), recent hospitalisation, colic surgery, or AMD therapy (*n* = 12), and peritonitis (*n* = 3). Twenty‐nine cases were excluded for unknown reasons, likely due to surgeon preference. One hundred and sixty horses were enroled in the study (Figure 1); 20 horses were excluded (24‐h group *n* = 9 and 72‐h group *n* = 11) for various reasons listed in Table S1. Therefore, a total of 140 horses completed the study, including 71 horses in the 24‐h and 69 horses in the 72‐h group. Study population data are described in Data S1.

Signalment and admission physical examination and clinicopathological variables for horses enroled in the 24‐h and 72‐h groups are shown in Table S2. The distribution of signalment data generally reflected the general population seen in our hospital,^5^ and there were no significant differences in mean bodyweight, sex, or breed across study groups. There were also no differences in physical exam or clinicopathologic variables across treatment groups. However, horses in the 72‐h group were significantly younger than horses in the 24‐h group (24‐h: 16 [9, 20] vs. 72‐h: 12 [6, 18] years). Surgery and anaesthetic variables are shown in Table S3. There was also no significant difference in any of these variables between SAP groups.

### 3.2 Postoperative complications

Postoperative complication data comparing the 24‐ and 72‐h SAP groups are presented in Table 1 (primary objective). There were no significant differences in the occurrence of any PO complications between the 24‐ and 72‐h SAP groups.

**TABLE 1:** Postoperative complications for horses enroled in a clinical trial comparing 24‐ and 72‐h surgical antimicrobial prophylaxis for colic surgery.

Variable	24‐h group	72‐h group	*p*‐value
Pyrexia—*N* (%; 95% CI)	39 (55%; 43%–66%)	42 (61%; 49%–72%)	0.5
Median (IQR) time from surgery to first pyrexia (h) [*N*]	12 (9, 24) [39]	12.5 (12, 50) [42]	0.3
Median (IQR) duration of pyrexia (h) [*N*]	6 (6, 56) [39]	12 (6, 26) [42]	0.6
Median (IQR) peak temperature (°C) [*N*]	36.6 (38.4, 39.3) [39]	38.6 (38.4, 39.1) [42]	0.7
Median (IQR) duration of peak temperature (h) [*N*]	6 (6, 6) [39]	6 (6, 6) [42]	0.7
Severity of pyrexia—*N* (%)			0.6
Slight (38.3–38.8°C)	24 (62%)	27 (64%)	
Mild (38.9–39.4°C)	6 (15%)	9 (21%)	
Moderate (39.5–39.9°C)	6 (15%)	5 (12%)	
Marked (≥40°C)	3 (8%)	1 (2%)	
Surgical site infection—*N* (%; 95% CI)	12 (17%; 10%–27%)	11 (16%; 9%–26%)	0.9
Median (IQR) time from surgery to diagnosis of surgical site infection (d) [*N*]	8 (6, 10)	8 (6, 10)	0.8
Catheter‐associated complications—*N* (%; 95% CI)	5 (7%; 3%–15%)	5 (7%; 3%–16%)	0.9
Median (IQR) time from surgery to diagnosis of catheter‐associated complications (d) [*N*]	8 (4, 10) [5]	4 (1, 6) [4]^a^	0.1
Pneumonia—*N* (%; 95% CI)	4 (6%; 2%–14%)	1 (1%; 0.2%–8%)	0.4
Median (IQR) time from surgery to diagnosis of pneumonia (d)[*N*]	2.5 (0.4, 4) [4]	14 [1]	0.1
Septic peritonitis—*N* (%; 95% CI)	3 (4%; 1%–12%)	1 (1%; 0.2%–8%)	0.6
Median (IQR) time from surgery to diagnosis of septic peritonitis (d) [*N*]	6 [0, 9]	10 [1]	0.4
Colic—*N* (%; 95% CI)	25 (35%; 25%–47%)	26 (38%; 27%–49%)	0.8
Median (IQR) time from surgery to signs of colic (d) [*N*]	3.5 (0.75, 8.5) [25]	2 (1, 5.25) [26]	0.5
Colic duration—*N* (%)			0.4
Transient <5 h	10 (40%)	15 (58%)	
6–24 h	4 (16%)	4 (15%)	
Multiple episodes	11 (44%)	7 (27%)	
Colic severity—*N* (%)			0.6
Mild	14 (56%)	14 (54%)	
Moderate	7 (28%)	10 (38%)	
Marked	4 (16%)	2 (8%)	
Relaparotomy—*N* (%)	2 (8%)	6 (23%)	0.1
Inappetence/anorexia—*N* (%; 95% CI)	15 (21%; 13%–32%)	8 (12%; 6%–21%)	0.1
Median (IQR) time from surgery to inappetence (h) [*N*]^b^	72 (48, 120) [13]	72 (24, 168) [5]	0.9
Duration of inappetence—*N* (%)			
Transient (≤24 h)	7 (46%)	2 (25%)	0.06
48 h	4 (27%)	0	
Prolonged (>48 h)	0 (0%)	2 (25%)	
Multiple intermittent periods	4 (27%)	4 (50%)	
Reflux—*N* (%; 95% CI)	7 (10%; 5%–19%)	10 (14%; 8%–25%)	0.4
Median (IQR) time from surgery to reflux (h) [*N*]	12 (6, 48) [7]	12 (0, 42) [10]	0.6
Median (IQR) duration of reflux (h) [*N*]	48 (19, 144) [7]	56 (30, 90) [10]	0.5
Median (IQR) peak rate reflux (L/h) [*N*]	3 (1.5, 4.5) [7]	3 (1, 5) [10]	0.6
Median (IQR) total volume reflux (L) [*N*]	67 (20, 95) [7]	36 (20, 83) [10]	0.5
Diarrhoea—*N* (%; 95% CI)	9 (13%; 7%–22%)	7 (10%; 5%–19%)	0.6
Median (IQR) time from surgery to diarrhoea (h) [*N*]	144 (42, 144) [9]	56 (12, 72) [7]	0.09
Median (IQR) duration of diarrhoea (h) [*N*]	48 (18, 222) [9]	36 (6, 144) [7]	0.6
Median (IQR) length of stay (d) [*N*]	7 (5, 10) [71]	7 (5, 9) [69]	0.2
Median (IQR) cost of treatment (US$) [*N*]	9805 (8533, 12,157) [71]	9702 (8484, 11,198) [69]	>0.9

*Note*: Abbreviations see Table S2.

^a^ One horse was diagnosed post‐discharge.

^b^ Five horses had multiple episodes of inappetence for which it was difficult to determine the time of onset.

Below is a description of the PO complications for *all cases* presented as *overall* median and IQR (25th and 75th percentiles) and, given that there was no significant association with the SAP group, data from both SAP groups were combined and variables that were significantly associated with each PO complication on multivariable analysis are described (secondary objective).

In total, 81 (58%) horses had PO pyrexia occurring at a median of 12 (12, 30) h after surgery and for a duration of 12 (6, 36) h. The median peak temperature of horses with pyrexia was 38.6 (38.4, 39.1)°C for a median of 6 (6, 6) h. Most PO pyrexia was classified as slight (51 [63]%), followed by mild (15 [18%]), moderate (11 [14%]), or marked (4 [5%]). Pyrexia was significantly associated with age (year, OR 0.92 [0.88–0.97], *p* < 0.001), PO reflux (OR [95% CI] 5.7 [1.2–26.5], *p* = 0.009), and SSI (3.4 [1.1–11.0], *p* = 0.03) on multivariable analysis.

Twenty‐three horses (16%) had an SSI with a median time from surgery of 8 (6, 10) days. Bacteria isolated from the SSI on culture are listed in Table S4. Surgical site infections were typically treated by maintaining cleanliness, providing adequate wound drainage, and abdominal bandage or hernia belt. Three horses with SSI also had septic peritonitis. Variables significantly associated with SSI in the multivariable model were surgeon category (Residents had 3.8 [1.3–10.8, *p* = 0.01] higher odds of SSI compared to Surgeon) and surgery duration (min, 9.1 [1.2–65.9]). Surgical site infection was also associated with repeat laparotomy (4 [50%] of horses undergoing relaparotomy had an SSI post relaparotomy whereas 19 [14%] without undergoing relaparotomy had an SSI, *p* = 0.02); septic peritonitis (3 [75%] horses with septic peritonitis and 20 [15%] without septic peritonitis had an SSI, *p* = 0.01) and PO reflux (7 [39%] horses with PO reflux and 16 [13%] without PO reflux had an SSI, *p* = 0.006); none were significant in a multivariable model likely because of small numbers and collinearity. No horse was euthanased specifically for SSI.

Ten (7%) horses had catheter‐associated complications with a median time from surgery to diagnosis of 5 (3, 8) days. Eight catheter‐associated complications were mild and managed by removing the IV catheter and applying topical diclofenac cream (Surpass®). Two horses with moderate septic thrombophlebitis were empirically treated with trimethoprim sulfamethoxazole. One horse grew *Actinobacillus equuli* subspecies equuili sensitive to all AMDs tested, and another horse had no growth. In a multivariable model, catheter‐associated complications were associated with bodyweight (kg, OR 0.98 [0.97–0.997], *p* = 0.009); IV fluid rate (L/h, OR 3.1 [1.07–9.22], *p* = 0.03); and IV fluid duration (h, OR 1.01 [1.0008–1.02], *p* = 0.04). Nine horses with catheter‐associated complications had pyrexia (*p* = 0.04). No horses were euthanased because of catheter‐associated complications.

Five horses had mild pneumonia with a median time from surgery to diagnosis of 4 (0.5, 9) days. Bacteria isolated from transtracheal wash samples (*n* = 2) were *Streptococcus equi* subspecies zooepidemicus (2) plus either *Enterobacter cloacae* and alpha‐haemolytic *Streptococcus* sp. (1) or *Bacillus* sp. and *Fusobacterium* sp. (1). *Streptococcus* sp. were sensitive to all indicated AMDs tested; *E. cloacae* was sensitive to aminoglycosides, tetracyclines, trimethoprim sulfamethoxazole, imipenem, chloramphenicol, and ceftazidime. Antimicrobial sensitivity on the other organisms was not obtained. Two horses were treated with trimethoprim sulfamethoxazole, two horses with potassium penicillin and gentamicin (one horse also with metronidazole). One horse with mild signs was not treated. Pneumonia was associated with anaesthesia duration (minute, OR 1.03 [1.01–1.05], *p* < 0.001) and timing of pre‐operative AMDs (minute, 1.07 [1.0–1.15], *p* = 0.049) on multivariable analysis. Four horses with pneumonia also had PO colic (*p* = 0.04). No horses were euthanased because of pneumonia.

Four horses had septic peritonitis, with a median time from surgery to diagnosis of 7.5 (1.5, 10) days. All four horses with septic peritonitis had PO pyrexia, and three horses (75%) had colic (*p* = 0.1), reflux (*p* = 0.007), and an SSI (*p* = 0.01). One horse was euthanased because of colic (no necropsy and possible ileal stump necrosis). Two horses developed septic peritonitis after relaparotomy. One horse had a light growth of *E. coli*, *Klebsiella pneumoniae*, *Enterobacter hormaechei*, and *Enterococcus faecium* from the peritoneal fluid and ventral midline incision. There was no AMD to which all organisms were sensitive, and the horse was treated with potassium penicillin, gentamicin, and metronidazole and discharged from the hospital alive. The other horse had a light growth of mixed gram‐positive and ‐negative organisms (*E. coli*, *E. faecalis*, *Lactobacillus* sp.) and mixed anaerobic growth from the peritoneal fluid (also had an SSI) and was euthanased. The horse diagnosed with septic peritonitis without any other complications and with no growth on bacterial culture was doing well at long‐term follow‐up.

Fifty‐one horses (36%) had at least one episode of colic after surgery, with a median time from surgery to colic onset of 48 (18, 144) h and most (25, 45%) of the horses having transient (<5 h) mild colic signs. Eight horses had a relaparotomy and were diagnosed with a segmental small intestinal volvulus (2), right dorsal displacement of the colon (2, one with concurrent fibrinous adhesions and one with inflammatory bowel disease), adhesions (2), NSLE (1), and impaction at the anastomosis (1). The only variable remaining in the multivariable model for PO colic was procedure category (horses undergoing SI R&A had 3.1 [1.37–7.02] higher odds of PO colic than horses undergoing exploratory laparotomy with repositioning only (*p* = 0.007) and 5.08 [1.58–16.37] higher odds than horses undergoing a pelvic flexure enterotomy with or without an enema (*p* = 0.006)).

Twenty‐three (16%) horses had a period of inappetence/anorexia. There were no clinically relevant variables associated with this non‐specific clinical finding.

Seventeen (12%) horses had PO reflux with a median time from surgery of 12 (5.5, 42) h, a duration of 48 (24, 108) h, a peak rate of 3 (1, 4.8) L/h, and a total volume of 52 (20, 87) L. Horses with PO reflux were diagnosed with ileus (15) or ileus and adhesions (2). Reflux was associated with the volume of admission reflux (L, OR 1.46 [1.15–1.84], *p* < 0.001) and lesion site (horses with SI lesions had 89.2 [2.16, 3680] higher OR of developing reflux compared to horses with LI lesions, *p* < 0.001). PO reflux was associated with PO colic (OR 8.15 [2.35–28.25], *p* < 0.001) and SSI (OR 5.57 [1.64–18.99], *p* = 0.006).

Sixteen (11%) horses had diarrhoea with a median time relative to surgery of 72 (5, 144) h, a duration of 48 (24, 180) h, and etiologies included *Clostridium difficile* (3), *C. perfringens* (1), and *Salmonella* sp. (2); however, in most horses, no etiological agent was identified. No variables were significantly associated with diarrhoea in multivariable analysis.

The median (IQR) length of stay (LOS) was 7 (5, 10) days, and the median cost of treatment was US$9724 (US$8527; US$11,611). Anaesthesia duration (*p* < 0.001) and the lowest MAP under anaesthesia (*p* = 0.01) were the only variables that predicted the expense of treatment. All complications increased the expense of treatment and LOS.

### 3.3 Shotgun metagenomic sequencing

Admission and discharge samples from 25 horses in the 24‐h group, 19 horses in the 72‐h group, and 5 medically managed horses (i.e., no antibiotics) underwent shotgun metagenomic sequencing. A total of 725,810,883 non‐host reads were generated, with an average (SD) of 9,180,012 (3,477,718) reads per sample. Positive and negative control samples were additionally sequenced to show cross‐contamination had not occurred (Figure S1). An average of 8.8% host reads were removed during quality control (Table S5). The distribution of horse and clinically relevant factors was similar among the three groups of horses. The mean (SD) length of stay (i.e., time from admission to discharge sample) of horses in each group was similar in the three groups: 5.2 (1.6) days in the 24‐h group, 5.3 (2.1) in the 72‐h group, and 5 (2) in the medically managed group.

**FIGURE 2:**
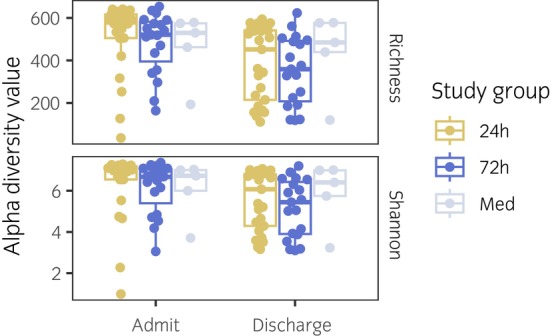
Alpha diversity of the gut microbiome of horses admitted for colic and receiving 24‐ or 72‐h of surgical antimicrobial prophylaxis (SAP) or no antibiotics (medically managed). Gold, 24‐h SAP; Blue, 72‐h SAP; Grey, medically managed (Med). Admit, admission.

The alpha diversity of the microbiome was significantly different between admission and discharge for all groups, with richness decreasing by 129 observed species (*p* = 0.002) and Shannon diversity decreasing by 0.8 units (*p* = 0.03) (Figure 2). However, there was no statistically significant difference in either metric between admission and discharge samples from the three groups (*p* = 1.0 for richness, *p* = 0.8 for Shannon).

**FIGURE 3:**
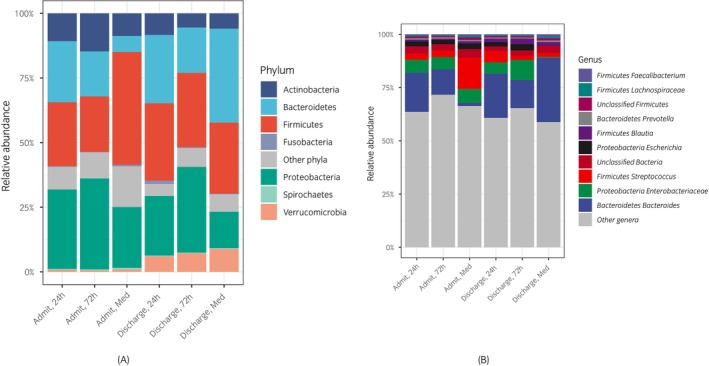
Admission (Admit) and discharge distribution of the most abundant phyla (A) and genera (B) in the faecal microbiome of horses admitted for colic and receiving 24‐ or 72‐h of surgical antimicrobial prophylaxis or no antibiotics (medically managed, Med).

Significant changes in the overall taxonomic composition of the microbiota occurred mostly over time, with timepoint (i.e., admission vs. discharge) being the only statistically significant variable on PERMANOVA (*p* = 0.001 for Bray‐Curtis distances, *p* = 0.01 for Jaccard distances). Non‐metric dimensional scaling (NMDS) plots showed the overlap among study groups' Bray‐Curtis distances (Figure S2). Among the individual taxa that had a mean relative abundance >1% across all samples (Figure S3), there were only a handful of taxa for which there was a borderline significant change in abundance between admission and discharge, including *Bacteroides thetaiotaomicron* (increase, *p* = 0.07), *Bacteroides uniformis* (increase, *p* = 0.2), *Bacteroides heparinolyticus* (increase, *p* = 0.2), and *Bacteroides dorei* (decrease, *p* = 0.2). Although no taxa exhibited statistically significant differences in relative abundance at discharge between groups, trends were observed suggesting a higher relative abundance of taxa typically associated with dysbiosis (e.g., *Escherichia* and other Enterobacteriaceae) in the 72‐h SAP group. Conversely, commensal genera generally regarded as beneficial, including *Bacteroides*, appeared reduced in the 72‐h SAP group (Figure 3).

**FIGURE 4:**
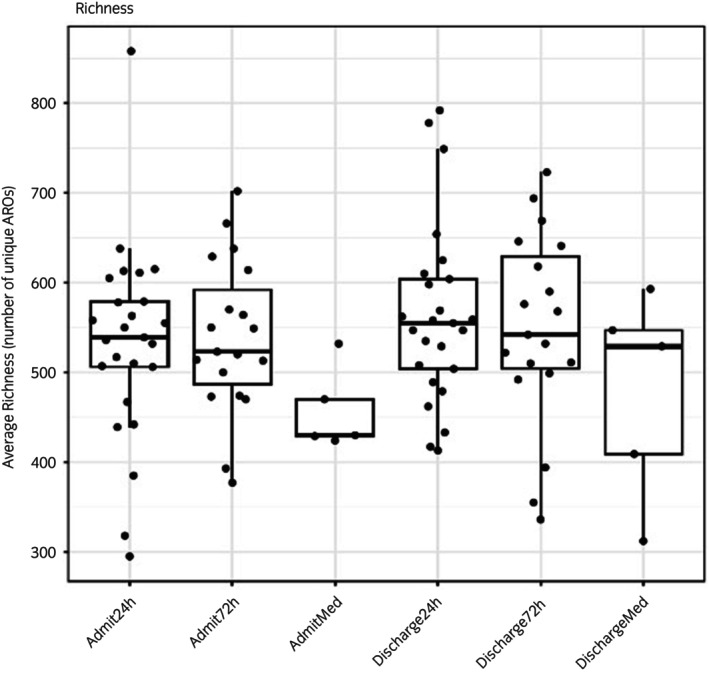
Admission (Admit) and discharge richness of antimicrobial resistance ontologies (AROs) in horses admitted for colic and receiving 24 h or 72 h of surgical antimicrobial prophylaxis or no antibiotics (medically managed, Med).

**FIGURE 5:**
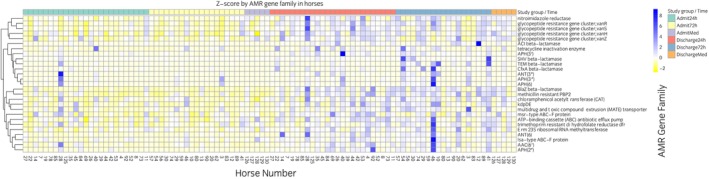
Heat map for admission (Admit) and discharge samples showing antimicrobial resistance gene families in faecal samples from horses admitted for colic and receiving 24 h or 72 h of surgical antimicrobial prophylaxis or no antibiotics (medically managed, Med).

There were no significant differences in the overall richness of AROs across any study groups (Figure 4). This was true even when ARO counts were rarified at various levels (Figure S4). Among the top 100 AMR gene families present in at least 99% of samples, there were 20 gene families that increased significantly in relative abundance between admission and discharge across all horses (Figure 5, Figure S5), including *macB*, *mel*, *evgS*, *arlR*, *arlS*, multiple *vanR* and *vanH* genes, *bcrA*, *smeR*, *lsaE*, *cdeA*, *kdpE*, and *Corynebacterium striatum tetA*. No statistically significant differences in levels of the most abundant AMR genes occurred between horses in the 24‐ and 72‐h SAP groups at discharge. Only one gene, *salA*, which confers resistance to lincosamides and streptogramins,^6^ was borderline significantly higher in the 72‐ than in the 24‐h SAP group at discharge (*p* = 0.07).

**FIGURE 6:**
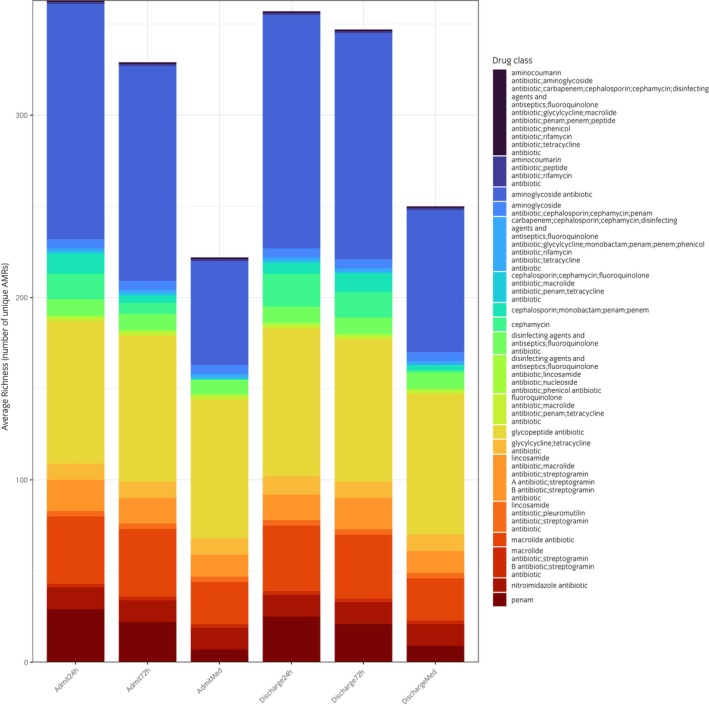
Changes in resistance to antimicrobial drug classes in horses admitted for colic and receiving 24 h or 72 h of surgical antimicrobial prophylaxis or no antibiotics (medically managed, Med). Admission (Admit) and discharge samples.

**FIGURE 7:**
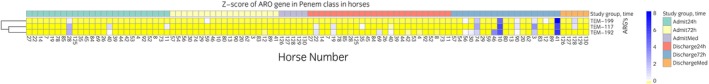
Heatmap of admission (Admit) and discharge samples showing TEM resistance genes in faecal samples from horses admitted for colic and receiving 24 h or 72 h of surgical antimicrobial prophylaxis or no antibiotics (medically managed, Med).

In contrast, levels of many AMR gene families increased between admission and discharge, particularly those associated with resistance to the administered antibiotics (penicillin and gentamicin, Figure 6). Specifically, genes associated with aminoglycoside‐modifying enzymes were significantly enriched in both treatment groups, including *AAC(6′)*, *ANT(3″)*, *ANT(6)*, *APH(2″)*, *APH(3′)*, *APH(3″)*, and *APH(6)* families, all of which confer resistance to gentamicin via enzymatic modification. Resistance genes targeting beta‐lactams were also significantly enriched, including canonical beta‐lactamase families such as *BlaZ*, *TEM*, *SHV*, and *CfxA*, as well as genes encoding methicillin‐resistant penicillin‐binding proteins (*PBP2*). While levels of most gene families increased to similar extents in both the 24‐ and 72‐h SAP groups, TEM beta‐lactamases, which confer resistance to penicillins and extended spectrum beta‐lactamases, were significantly higher in the 72‐ compared to the 24‐h SAP group at discharge (*p* = 0.003, Figure 7). We conducted a sensitivity analysis using two different techniques and obtained consistent results (Figure S6).

## 4 DISCUSSION

In this prospective, randomised clinical trial comparing 24‐ and 72‐h SAP for exploratory laparotomy for colic, there were no significant or clinically relevant differences in GI‐ or infection‐related complications between SAP groups. Randomisation was deemed adequate, with similar numbers in each group and no significant difference in clinical variables between groups except for patient age. Age was not associated with any complications. Notably, all cases were considered for inclusion in this study rather than only clean or select clean‐contaminated exploratory laparotomy procedures^21^ albeit some clinicians' reasons for deciding not to include a case were ill‐defined. A priori exclusion criteria were used to reduce confounding variables that may have increased or decreased complications or affected the microbiome or resistome.

Previous studies comparing different durations of SAP for horses undergoing colic surgery have had mixed results. Most previously published prospective and retrospective studies found no statistically significant difference in SSI rates between horses receiving different durations of SAP.^17^, ^18^, ^19^, ^20^, ^22^, ^23^, ^24^ However, one study^21^ found that horses administered SAP only pre‐and intra‐operatively had higher rates of SSI (in‐hospital 16% and at 4 weeks 22%) than horses administered 72 h of SAP (in‐hospital 0% and 3% at 4 weeks). While a significant difference was not observed in another prospective study^20^ comparing a single pre‐operative dose with a 5‐day SAP regimen, horses in the single‐dose group had an SSI of 23% compared with 5% for horses receiving a 5‐day regimen. Both these studies had small numbers and only compared the single and/or intraoperative dose to 3 or 5 days of SAP. Notably, an older study found no difference in SSI rates between a 3 and 5‐day SAP regimen.^17^ In another retrospective study,^23^ horses receiving SAP ≤12 h had a 30% infection rate compared to horses receiving 13–24 h (13%), 25–48 h (18%), and 29–72 h (19%); albeit the difference did not reach statistical significance. Horses in the latter study^23^ receiving >72 h SAP had an infection rate of 27%^23^; however, that may have been confounded by surgeon choice based on the procedure performed and other perioperative variables.^24^ While definitive conclusions about single‐dose or two‐dose SAP (i.e., pre‐operatively or pre‐ and intraoperatively) cannot really be made based on these studies because of the aforementioned limitations, it is possible that horses may need extended SAP beyond recovery from general anaesthesia. Findings from previous studies combined with those from the current study suggest that horses may need SAP for 24 h; however, beyond that time SAP is likely unnecessary. Performing a large multicentre randomised clinical trial comparing peri‐operative only to 24‐h SAP would be a next step to draw firm conclusions about SAP duration <24 h.

Several variables other than SAP duration were associated with PO complications. Most of these variables have been associated with infection in previous studies or have a logical association with the development of infection. Surgeon experience^32^ and surgery duration^33^, ^34^, ^35^ were associated with SSI, as was found in previous studies. Duration of IV fluid administration (and by extrapolation jugular vein catheterisation^36^, ^37^) and the rate of IV fluid administration were associated with catheter‐associated complications. Some of these clinical variables associated with complications, specifically infection, may be modified by means other than SAP duration, which is vital given the importance of AMR.

Most horses had a mild transient PO pyrexia. These findings are similar to another study^22^ where 85% of horses had PO pyrexia after colic surgery. In the previous study,^22^ while the presence of PO pyrexia (any rectal temperature >38.3°C) was not associated with infection, specific pyrexia details, namely time to first pyrexia and peak temperature >48 h after surgery, peak temperature >39.2°C, and duration of pyrexia >48 h were associated with infection. While this was not specifically evaluated in the current study, our findings support the earlier study in that horses often have mild transient pyrexia early during the PO period, which is not always predictive of infection. This is particularly the case given that pyrexia was also associated with age and PO reflux as well as SSI in our study.

This study also gave us the unique opportunity to examine the effect of hospitalisation and different durations of SAP on the equine faecal microbiome and resistome. As was found in other studies examining the microbiome of hospitalised horses,^38^, ^39^ we found that hospitalisation itself was one of the most important drivers of microbiome change. Horses in both SAP groups and in the medically managed group experienced significant decreases in alpha diversity over time as well as decreases in levels of beneficial commensals such as *Bacteroides* species and increases in pathogenic bacteria such as *Escherichia*, as was seen elsewhere.^39^ However, the fact that SAP horses experienced more pronounced changes than the medically managed horses indicates that AMDs also had a strong effect on the microbiome and resistome. While there were no statistically significant differences in levels of these taxa between groups, trends indicated a more pronounced effect in the 72‐ than in the 24‐h group. Similarly, while there were no statistically significant differences in the overall richness and diversity of AMR genes over time or between groups, levels of aminoglycoside and beta‐lactam resistance genes increased over time and were significantly higher in the 72‐h group at discharge than in the 24‐h group, indicating substantial selection pressure.

It is unknown whether these increases in AMR genes would necessarily result in increased carriage of or infection with phenotypically resistant pathogens. The AMR genes that increased following surgery, particularly those conferring resistance to beta‐lactams (e.g., *blaZ*, *TEM*, *SHV*, *CfxA*) and aminoglycosides (e.g., *aac(6′)*, *aph(3′)*, *ant(6)*), are of potential clinical concern. Many of these genes are plasmid‐borne and have been documented to transfer horizontally between bacterial species in horses.^40^, ^41^ Additionally, plasmid‐mediated *bla_TEM* and aminoglycoside resistance genes have been identified in MDR *E. coli* and *Klebsiella pneumoniae* isolates from hospitalised horses.^2^, ^19^ These genes often reside on conjugative plasmids which facilitate the co‐transfer of multiple resistance determinants across diverse bacterial hosts,^42^, ^43^, ^44^ especially in the context of inflammation^45^ such as that experienced during colic and surgery. The expansion of such genes in the GI microbiome raises concern for future opportunistic infections in horses, particularly when considering the Trojan‐horse mechanism of SSIs,^46^, ^47^ whereby sterile postoperative wounds become infected by a pathogen originating from a remote site such as the GI tract. Notably, the pathogens that caused SSIs in the horses of our trial were frequently multidrug resistant (Table S4).

Our study suggests that 24 h of SAP is preferable, as similar rates of PO complications and better microbiome/resistome outcomes were achieved with the shorter duration. However, we acknowledge that the lack of a statistically significant difference is not the same as true non‐inferiority, and it is possible our study was simply not powered to detect a difference in infection rates smaller than the 15% difference we were powered to detect. Very large sample sizes are often needed to demonstrate true non‐inferiority of two protocols (i.e., very small detectable differences, often <1%), which would not have been feasible to recruit in our hospital. As is often the case with clinical trials that end up taking much longer and being more expensive than anticipated,^48^ we ended up needing almost three times the projected timeframe to enrol our target number of patients. However, multiple studies have demonstrated similar post‐operative outcomes in patients receiving shorter vs. longer durations of SAP.^17^, ^18^, ^19^, ^20^, ^22^, ^23^, ^24^ Thus, our study adds to the layers of evidence suggesting that a shorter duration is as effective and safer from a microbiological point of view. From a clinically practical point of view, 24 h is also preferred, as it limits the time needed for an intravenous catheter. Anecdotally, clinicians at our hospital became frustrated having horses in the 72‐h SAP group because it prolonged the duration of intravenous catheterisation; and while SAP group was not statistically significantly associated with catheter‐related complications (most likely due to the small number of such complications), there is at least a perception among clinicians that such an association exists.

Limitations of our study include the possibility of reduced generalisability since the study was performed at a single institution. However, we believe that the large number of surgeons (14) that were involved in the study provides some mitigation against that possibility. The lack of blinding of clinicians to the treatment allocation of their patients may also have resulted in information bias, but we attempted to limit this possibility by having objectively measured PO outcomes. Finally, horses in the 72‐h group tended to be younger than horses in the 24‐h group, which could have resulted in confounding of the outcomes. Despite these limitations, our results suggest that SAP beyond 24 h is unnecessary to prevent complications following colic surgery. Additionally, shorter durations have the benefit of inducing less AMR selective pressure and being more clinically practical for patient management.

## FUNDING INFORMATION

The research was funded by the Raymond Firestone Research Foundation.

## CONFLICT OF INTEREST STATEMENT

The authors declare no conflicts of interest.

## AUTHOR CONTRIBUTIONS

**Louise L. Southwood:** Conceptualization; investigation; writing – original draft; methodology; formal analysis; project administration; supervision; data curation; writing – review and editing. **Alicia Long:** Investigation; writing – review and editing; data curation. **Jairo Perez:** Investigation; writing – review and editing; data curation. **Scott Daniel:** Investigation; writing – original draft; methodology; software; formal analysis. **Kyle Bittinger:** Methodology; formal analysis; software. **Maia Aitken:** Funding acquisition; writing – review and editing. **Laurel Redding:** Funding acquisition; writing – review and editing; writing – original draft; methodology; data curation; formal analysis.

## DATA INTEGRITY STATEMENT

Louise L. Southwood (clinical data) and Laurel Redding (microbiome and resistome data) had full access to all the data in the study and take responsibility for the integrity of the data and the accuracy of the data analysis.

## ETHICAL ANIMAL RESEARCH

The study was approved by the Institutional Animal Care and Use Committee (Protocol 807182).

## INFORMED CONSENT

Informed consent was obtained for study inclusion.

## Supporting information


**Data S1.** Description of study population signalment and clinical variables.

**Figure S1.** Heatmap of bacterial taxa across time points and groups, including positive and negative control samples.

**Figure S2.** NMDS plots showing resistome composition at admission and discharge. We performed non‐metric multidimensional scaling (NMDS) to complement the Principal Coordinate Analysis (PCoA) in the original manuscript. Unlike PCoA, NMDS does not assume linear relationships and is well suited for visualising community composition based on rank‐order dissimilarity. Each point represents a sample, and distances between points reflect differences in antimicrobial resistance gene profiles, calculated using Bray–Curtis dissimilarity. Convex hulls group samples by study day (24 h, 72 h, or Med) to illustrate within‐group variation. (A) NMDS of samples collected at hospital admission. (B) NMDS of samples collected at discharge. These plots allow visualisation of how resistome profiles vary between timepoints and among participants within each phase of hospitalisation.

**Figure S3.** Taxa with a mean relative abundance >1% across all samples.

**Figure S4.** Comparison of ARO richness across timepoints using three rarefaction approaches. Richness is shown for each timepoint using three different methods to account for variation in sequencing depth: (A) no rarefaction, (B) rarefaction to the median number of reads per sample (37,045), and (C) rarefaction to the minimum number of reads per sample (2218). Rarefaction involves subsampling to a uniform depth to allow fair comparisons across samples. As expected, richness values decrease with lower rarefaction thresholds, but overall trends across timepoints remain consistent.

**Figure S5.** Antimicrobial resistance gene families and ontologies.

**Figure S6.** Abundance of TEM beta‐lactamase genes at discharge in the 24 h group vs. the 72 h group. Panel A shows a heat map of three specific TEM beta‐lactamase genes (TEM‐117, TEM‐126, and TEM‐192) across patient samples at admission and discharge in the 24 h and 72 h group. Colour intensity reflects gene abundance after adding a small pseudocount to handle undetected genes (i.e., zero values), with higher expression shown in dark blue and lower expression in yellow/white. Rows represent genes, and columns represent individual patient samples, grouped by timepoint and group. Panel B shows the results of a zero‐inflated beta regression model that accounts for the large number of samples where specific genes were not detected. This model estimates the probability that each gene was increased or decreased relative to its level in the 24 h group. Points to the right of the dashed line indicate increased abundance in the 72 h group compared to the 24 h group. Filled circles denote statistically significant changes. TEM‐126 and TEM‐117 were significantly more abundant in the 72 h group, while TEM‐192 was not significantly different.

**Table S1.** Specific reasons for exclusion for each horse.

**Table S2.** Signalment, physical exam, and clinicopathologic variables for horses enroled in a clinical trial comparing 24‐ and 72‐h of surgical antimicrobial prophylaxis for colic surgery.

**Table S3.** Surgical/general anaesthesia and post‐operative treatment variables for horses enroled in a clinical trial comparing 24‐ and 72‐h of surgical antimicrobial prophylaxis for colic surgery.

**Table S4.** Bacteria isolated on culture from the incisional infection.

**Table S5.** Percentage of host‐derived sequencing reads removed during quality control processing. This table shows the proportion of raw sequencing reads that were identified as host DNA (e.g., horse) and removed during quality control filtering prior to downstream metagenomic analysis. Removing host reads is a standard step to ensure analysis focuses on microbial DNA.

## Data Availability

Raw data are available under BioProject PRJNA1308598. Code and statistics to generate all figures and reports are available here: https://doi.org/10.5281/zenodo.17476471. The clinical data that support the findings of this study are available upon reasonable request from the corresponding author. Open clinical data sharing exemption granted by the editor.
