# Supplementary material for: Effect of surgical antimicrobial prophylaxis duration for colic surgery on complications and resistome
Source: Equine Vet J. 2025 Dec 10;58(2):390–403. doi: 10.1002/evj.70137 (PMC12892381; doi:10.1002/evj.70137)
Supplement: Supplementary file 1 — Data S1. Description of study population signalment and clinical variables. [file EVJ-58-390-s008.pdf]

Equine Veterinary Journal BEVA

Data S1: Study population signalment and clinical variables.

The median (25th, 75th percentile) age of horses was 14 (7, 19) years. Most horses were categorised as American breeds (44, 31%), Thoroughbred (36, 26%), Warmblood (26, 19%), or Standardbred (20, 14%), with smaller numbers of crossbreeds (N=6), Arabian horses (5), and other breeds (3). There were 79 (56%) geldings, 52 (37%) females, and 9 intact males (6%)

The median bodyweight of horse was 530 (480, 584) kg. Median colic duration was 7 (4, 17) hours. Median admission physical examination findings were rectal temperature 99.3 (98.2, 99.5)°F, heart rate 52 (44, 60) beats/minute, respiratory rate 20 (16, 24) breaths/minute. Mucus membranes were pink/tacky in 65 (47%) and pink/moist in 55 (40%) of horses with 15 (11%) horses having injected/tacky and 2 horses having dark red/dry mucus membranes. Capillary refill time was <2 seconds in 52 (50%) horses, 2-2.5 seconds in 34 (33%), and 3-4 seconds in 18 (17%) horses. Most horse did not have reflux on nasogastric intubation (median and 25th, 75th 0L). Media admission laboratory data were PCV 0.40 (0.35, 0.44)L/L, TS 7 0(63, 74) g/L, blood lactate concentration 1.6 (0.9, 2.8) mmol/L, blood glucose concentration (9.4 (7.2, 11.7) mmol/L, creatinine concentration (118 (100, 139) mmol/L, plasma ionised calcium 1.39 (1.31, 1.46) mmol/L, plasma sodium 136 (134, 138) mEq/L, plasma chloride (98 (95, 100) mEq/L, plasma potassium (3.4 (3.2, 3.7) mEq/L. Peritoneal fluid analysis was performed in 71 horses and the colour was described as serosanguineous (32, 45%) or yellow (30, 42%) in most horses with 6 horses having yellow/orange, 2 horses having an enterocentesis, and 1 horse having haemorrhagic fluid. Median peritoneal fluid TS 30 (37, 20) g/L and lactate concentration 4 (2.9, 6) mmol/L. Peritoneal nucleated cell count was only measured in 6 horses (1.44 [1.10, 58.0] x10^9 cells/uL).

Horses received perioperative antimicrobials at a median of 15 (10, 25) minutes prior to the start of surgery. Median anesthesia duration was 175 (135, 225) minutes and surgery duration 135 (91, 185) minutes. The lowest recorded MAP was 66 (62, 70) mmHg, lowest PaO_2 163 (93, 301) mmHg, PaCO2 (53, 50, 59) mmHg. Intraoperative PCV was measured in 119 horses with a median of 0.40 (0.38, 0.45) L/L, total solids in 113 horses with a median of 54 (50, 58) g/L, and blood lactate concentration in 94 horses with a median of 1.75 (1.3, 2.4) mmol/L. Seventy-seven (55%) horses had SI, 60 (43%) LI lesions and 3 horses were described as having LI/SI lesions (e.g. ileus). Ninety-eight (70%) had a strangulating and 42 (30%) had a non-strangulating obstruction. Lesion diagnoses were strangulation of the SI or small colon by a pedunculated lipoma (38, 27%), large colon volvulus (26, 19%), other small intestinal strangulation (11, 8%), large or small colon impaction (9, 6%), right dorsal displacement of the large colon (8, 6%), small intestinal volvulus (8, 5%), epiploic foramen entrapment (7, 5%), left dorsal displacement of the large colon with or without nephrosplenic entrapment (NSLE, 7, 5%), ileal or jejunal impaction (6, 4%), adhesions (6, 4%), gastrosplenic ligament entrapment (4, 2%), and fecalith (3), other large intestinal strangulation (2), ileus (2), , enterolithiasis (1), muscular hypertrophy (1) and open diagnosis (1). Exploratory laparotomy with repositioning of the intestine was performed in 64 horses (46%), small intestinal resection and anastomosis in 41 horses (29%), pelvic flexure enterotomy with or without an

enema in 23 horses (16%) and 6 horses had another large intestinal enterotomy/enterectomy and 6 horses had a procedure classified as other (e.g. adhesiolysis). Sixty-one horses were re-dosed with potassium penicillin one time and 7 horses were re-dosed twice during general anaesthesia. Most horses (111, 79%) had an Ioban® used for wound protection, 20 (14%) horses had a stent plus an Ioban®, and 9 (7%) horses had a stent only. Median recovery duration was 55 (70, 40) minutes and only 7 horses had their wound protection dislodged. An abdominal bandage was placed on 61 (44%) horses. Most cases were operated on by an experienced ACVS Diplomate (90, 63%) with 27 (19%) by an ACVS resident and 25 (18%) by a junior ACVS or ACVECC Diplomate.

The median duration of treatment with flunixin meglumine was 10 (84, 120) hours. Most horses (116, 83%) were treated with IV lidocaine with a median duration of 29 (20, 44) hours. The median IV fluid rate was 1.5 (1, 2) L/h with a median duration of 28 (20, 40) hours and most horses were supplemented with KCl (61, 45%), KCl and calcium gluconate (20, 15%), KCl, calcium gluconate and dextrose (9, 6%), or some combination of KCl, calcium gluconate, dextrose, magnesium, and thiamine (17 (12%). Thirty-three (24%) horses had no supplementation. The overall time to first feed relative to surgery was 24 (12, 24) hours with most horses being fed Equine Senior complete feed pellets (Purina or Triple Crown, 87, 62%) or alfalfa hay (32, 23%). Other feeds were timothy hay (7), grass or grass plus Equine Senior (6), Equine Senior and alfalfa hay (4), Equine Senior and timothy hay (2), and dengi hay or other feed (2). Median time to full feed was 120 (84, 160.5) hours with most horses being fed free-choice mixed timothy and alfalfa hay with Equine Senior (45, 33%), free-choice mixed timothy and alfalfa hay alone (40, 29%), timothy hay and Equine Senior (19, 13%), timothy hay (10, 7%), alfalfa hay and Equine Senior (7, 5%), and alfalfa hay alone (7, 5%). Eight horses were fed other feed including Equine Senior pellets as a complete feed, dengie hay, or hay pellets. Four horses were not of full feed at the time of hospital discharge or euthanasia.
