# Supplementary material for: Effect of surgical antimicrobial prophylaxis duration for colic surgery on complications and resistome
Source: Equine Vet J. 2025 Dec 10;58(2):390–403. doi: 10.1002/evj.70137 (PMC12892381; doi:10.1002/evj.70137)
Supplement: Supplementary file 3 — Figure S2. NMDS plots showing resistome composition at admission and discharge. We performed non‐metric multidimensional scaling (NMDS) to complement the Principal Coordinate Analysis (PCoA) in the original manuscript. Unlike PCoA, NMDS does not assume linear relationships and is well suited for visualising community composition based on rank‐order dissimilarity. Each point represents a sample, and distances between points reflect differences in antimicrobial resistance gene profiles, calculated using Bray–Curtis dissimilarity. Convex hulls group samples by study day (24 h, 72 h, or Med) to illustrate within‐group variation. (A) NMDS of samples collected at hospital admission. (B) NMDS of samples collected at discharge. These plots allow visualisation of how resistome profiles vary between timepoints and among participants within each phase of hospitalisation. [file EVJ-58-390-s007.pdf]

Equine Veterinary Journal BEVA

Figure S2: NMDS plots showing resistome composition at admission and discharge.

We performed non-metric multidimensional scaling (NMDS) to complement the Principal Coordinate Analysis (PCoA) in the original manuscript. Unlike PCoA, NMDS does not assume linear relationships and is well suited for visualising community composition based on rank-order dissimilarity.

Each point represents a sample, and distances between points reflect differences in antimicrobial resistance gene profiles, calculated using Bray–Curtis dissimilarity. Convex hulls group samples by study day (24h, 72h, or Med) to illustrate within-group variation.

A) NMDS of samples collected at hospital admission
B) NMDS of samples collected at discharge

These plots allow visualisation of how resistome profiles vary between timepoints and among participants within each phase of hospitalisation.

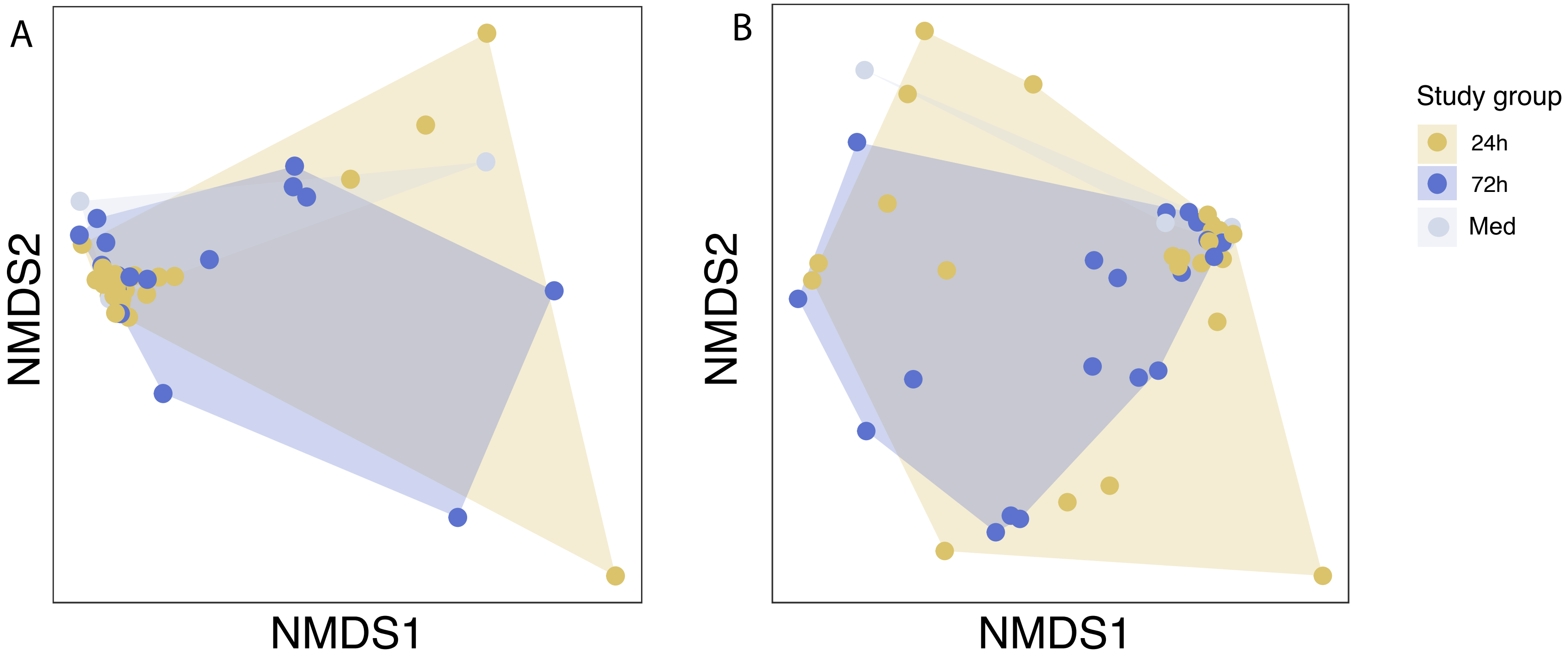
A
NMDS2
NMDS1
B
NMDS2
NMDS1
Study group
24h
72h
Med
