# Supplementary figures and images for: Effect of surgical antimicrobial prophylaxis duration for colic surgery on complications and resistome
Source: Equine Vet J. 2025 Dec 10;58(2):390–403. doi: 10.1002/evj.70137 (PMC12892381; doi:10.1002/evj.70137)

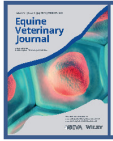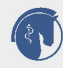

**Figure S3:** Taxa with a mean relative abundance >1% across all samples.

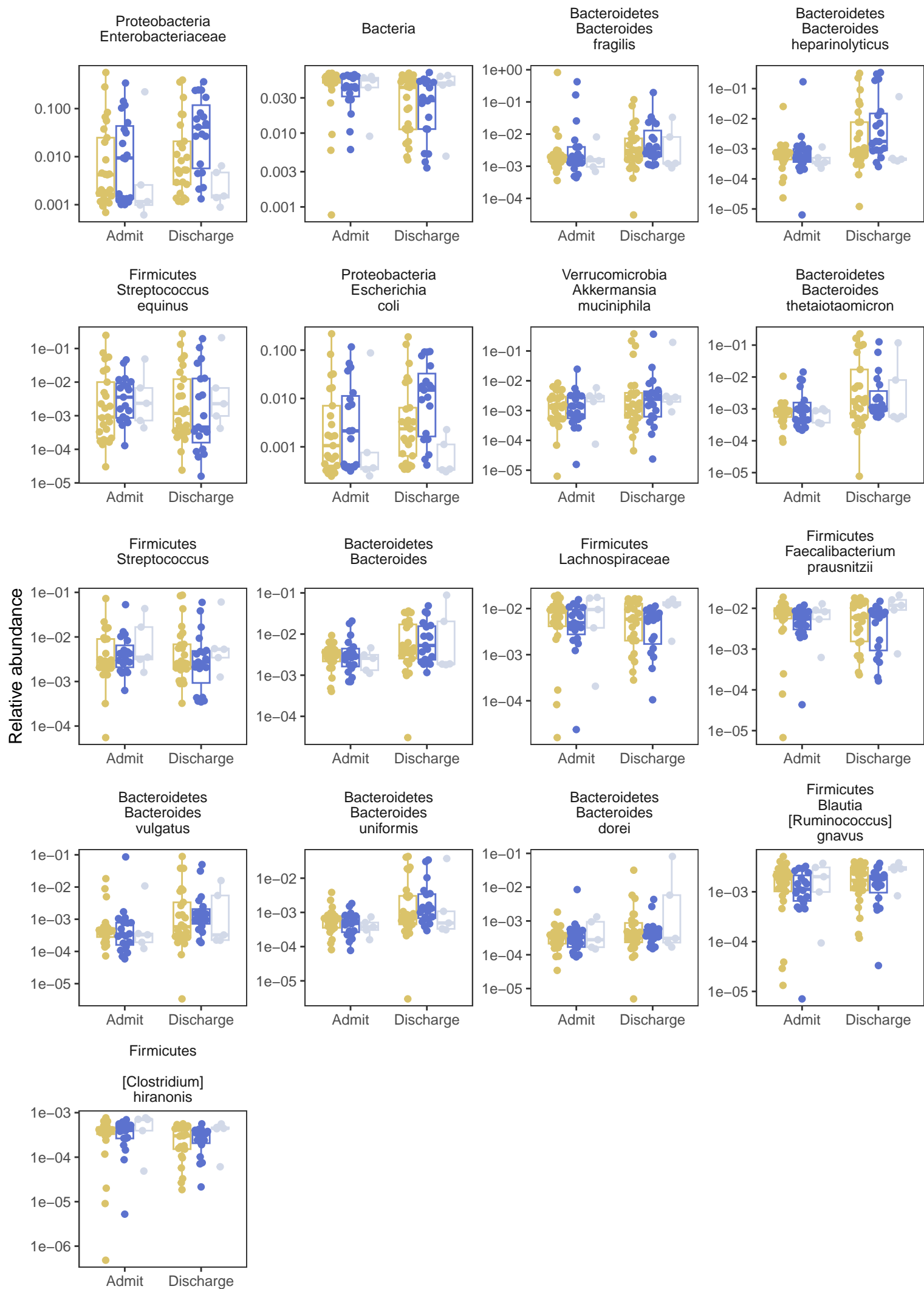

Supplement: Supplementary file 4 — Figure S3. Taxa with a mean relative abundance >1% across all samples. [file EVJ-58-390-s003.pdf]
