# Supplementary material for: Effect of surgical antimicrobial prophylaxis duration for colic surgery on complications and resistome
Source: Equine Vet J. 2025 Dec 10;58(2):390–403. doi: 10.1002/evj.70137 (PMC12892381; doi:10.1002/evj.70137)
Supplement: Supplementary file 5 — Figure S4. Comparison of ARO richness across timepoints using three rarefaction approaches. Richness is shown for each timepoint using three different methods to account for variation in sequencing depth: (A) no rarefaction, (B) rarefaction to the median number of reads per sample (37,045), and (C) rarefaction to the minimum number of reads per sample (2218). Rarefaction involves subsampling to a uniform depth to allow fair comparisons across samples. As expected, richness values decrease with lower rarefaction thresholds, but overall trends across timepoints remain consistent. [file EVJ-58-390-s010.pdf]

Figure S4: Comparison of ARO richness across timepoints using three rarefaction approaches. Richness is shown for each timepoint using three different methods to account for variation in sequencing depth:
A) no rarefaction, B) rarefaction to the median number of reads per sample (37,045), and C) rarefaction to the minimum number of reads per sample (2,218).
Rarefaction involves subsampling to a uniform depth to allow fair comparisons across samples. As expected, richness values decrease with lower rarefaction thresholds, but overall trends across timepoints remain consistent.

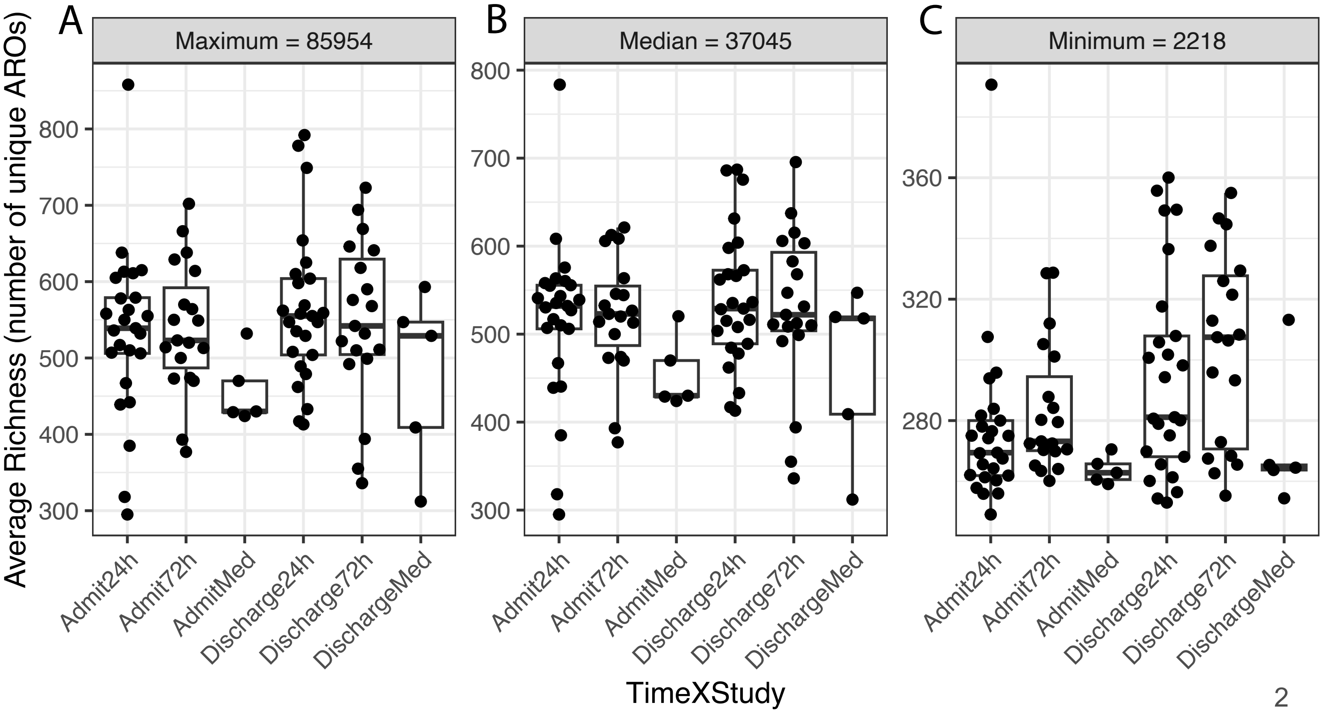
A
Maximum = 85954
B
Median = 37045
C
Minimum = 2218
Average Richness (number of unique AROs)
800
700
600
500
400
300
360
320
280
Admit24h
Admit72h
AdmitMed
Discharge24h
Discharge72h
DischargeMed
TimeXStudy
