# Supplementary material for: Effect of surgical antimicrobial prophylaxis duration for colic surgery on complications and resistome
Source: Equine Vet J. 2025 Dec 10;58(2):390–403. doi: 10.1002/evj.70137 (PMC12892381; doi:10.1002/evj.70137)
Supplement: Supplementary file 6 — Figure S5. Antimicrobial resistance gene families and ontologies. [file EVJ-58-390-s012.pdf]

Equine Veterinary Journal BEVA

Figure S5: Antimicrobial resistance gene families and ontologies.

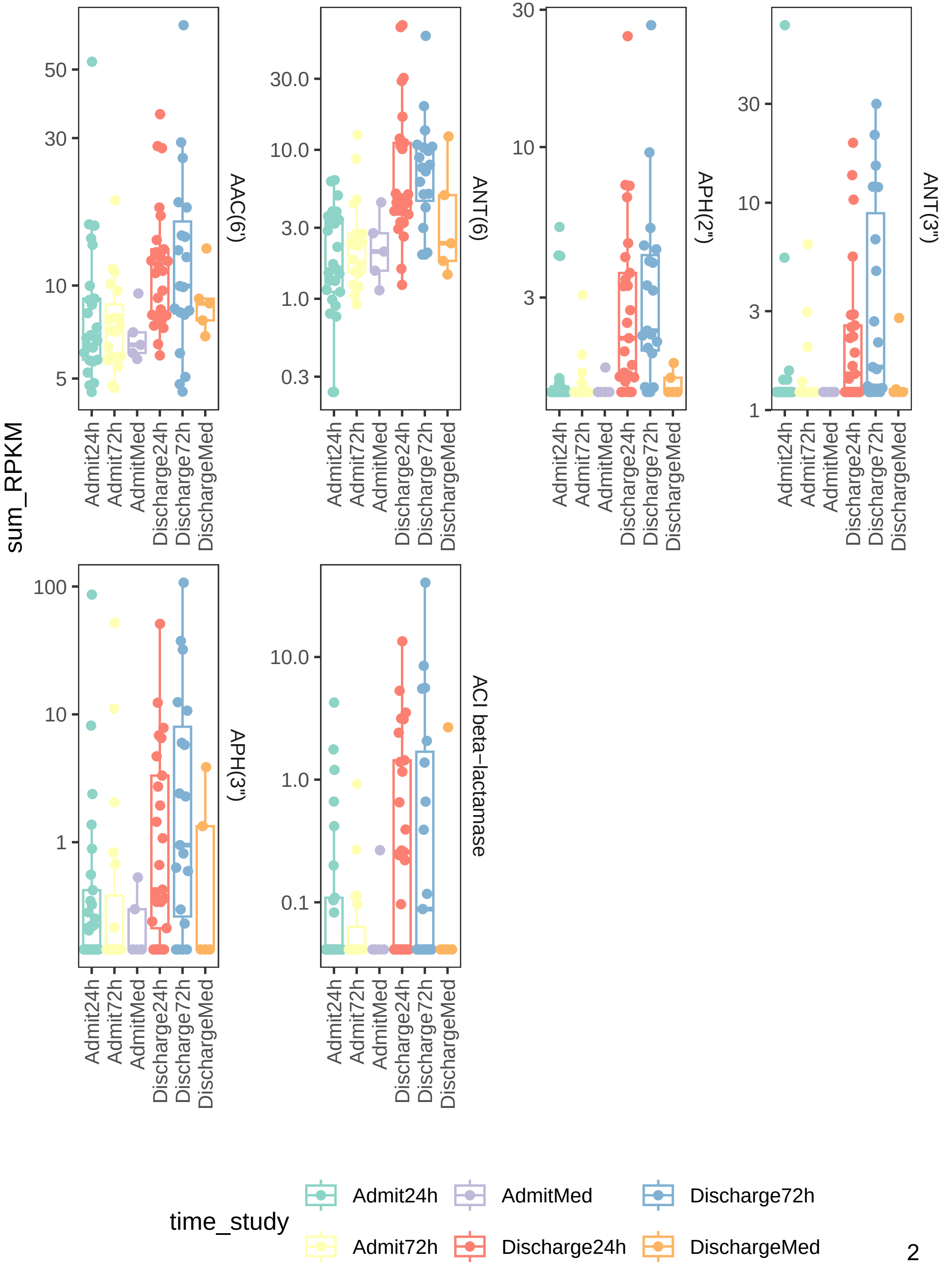

sum_RPKM
AAC(6')
ANT(6)
APH(2")
ANT(3")
APH(3")
ACI beta−lactamase
Admit24h
Admit72h
AdmitMed
Discharge24h
Discharge72h
DischargeMed
time_study

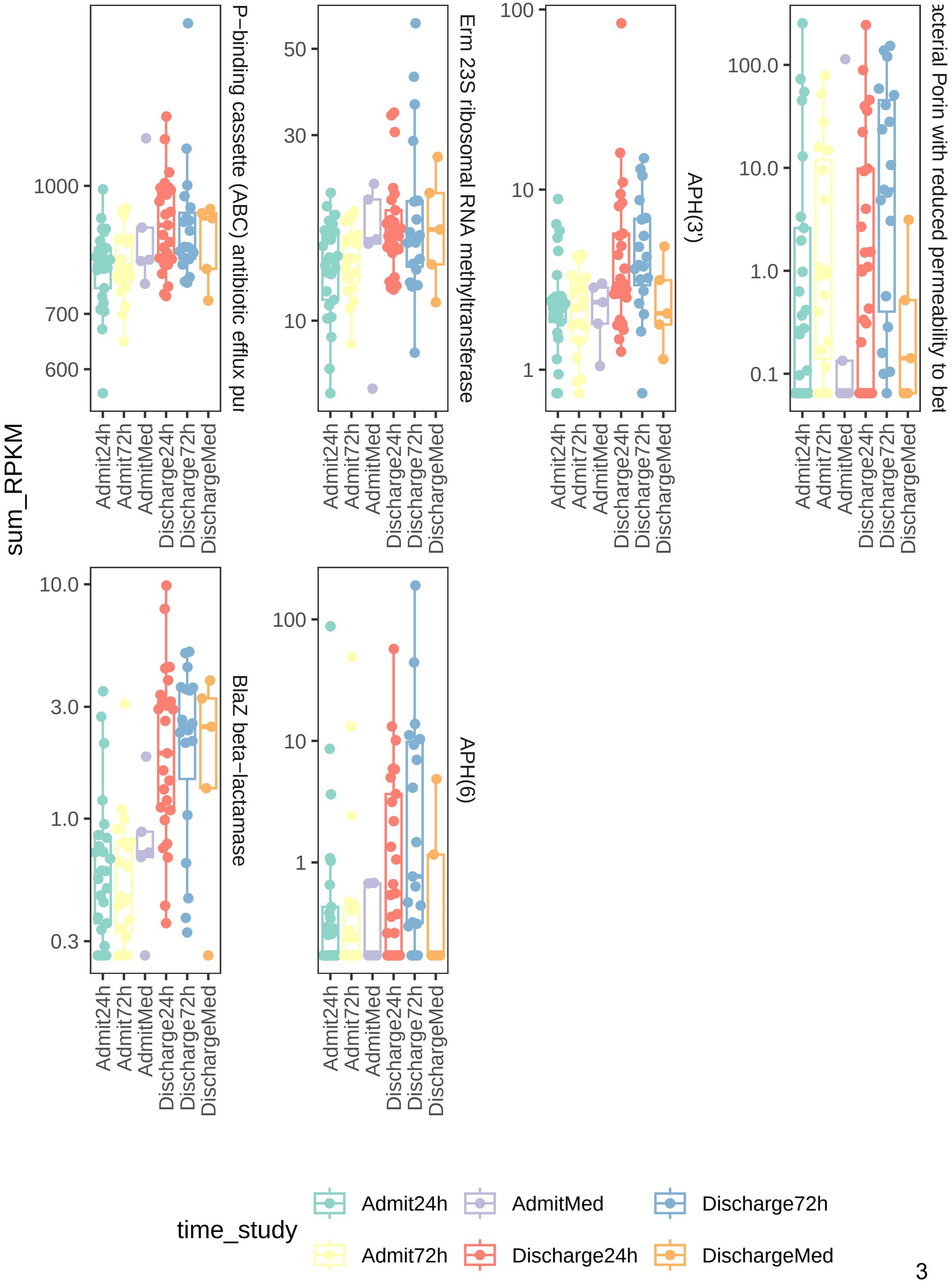
sum_RPKM
P–binding cassette (ABC) antibiotic efflux pur
Erm 23S ribosomal RNA methyltransferase
APH(3')
acterial Porin with reduced permeability to bet
BlaZ beta–lactamase
APH(6)
1000
700
600
50
30
10
100
10
1
100.0
10.0
1.0
0.1
10.0
3.0
1.0
0.3
100
10
1
Admit24h
Admit72h
AdmitMed
Discharge24h
Discharge72h
DischargeMed
time_study
Admit24h
Admit72h
AdmitMed
Discharge24h
Discharge72h
DischargeMed

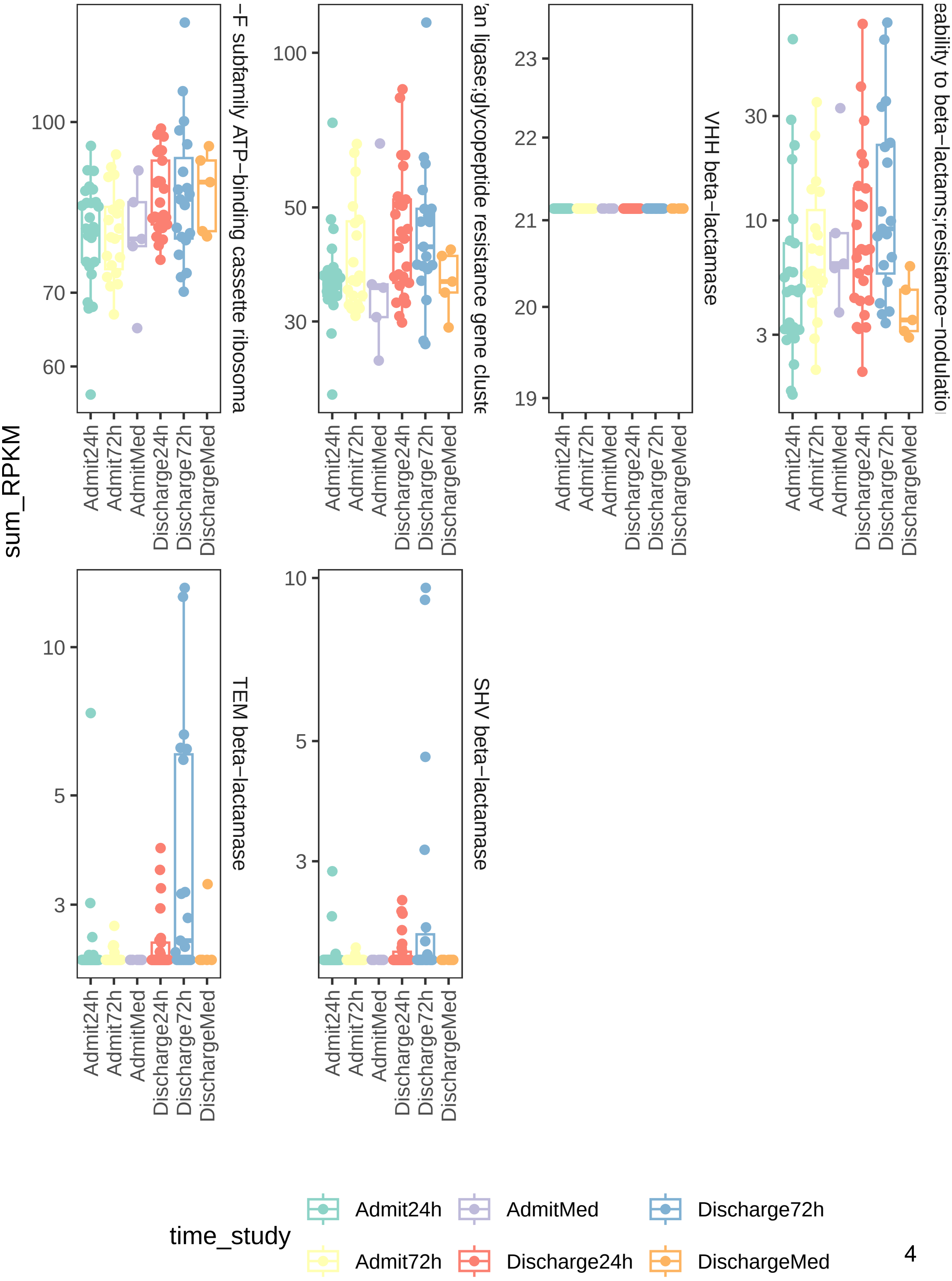

sum_RPKM
–F subfamily ATP–binding cassette ribosoma
'an ligase;glycopeptide resistance gene cluste
VHH beta–lactamase
eability to beta–lactams;resistance–nodulatio
TEM beta–lactamase
SHV beta–lactamase
Admit24h
Admit72h
AdmitMed
Discharge24h
Discharge72h
DischargeMed
time_study

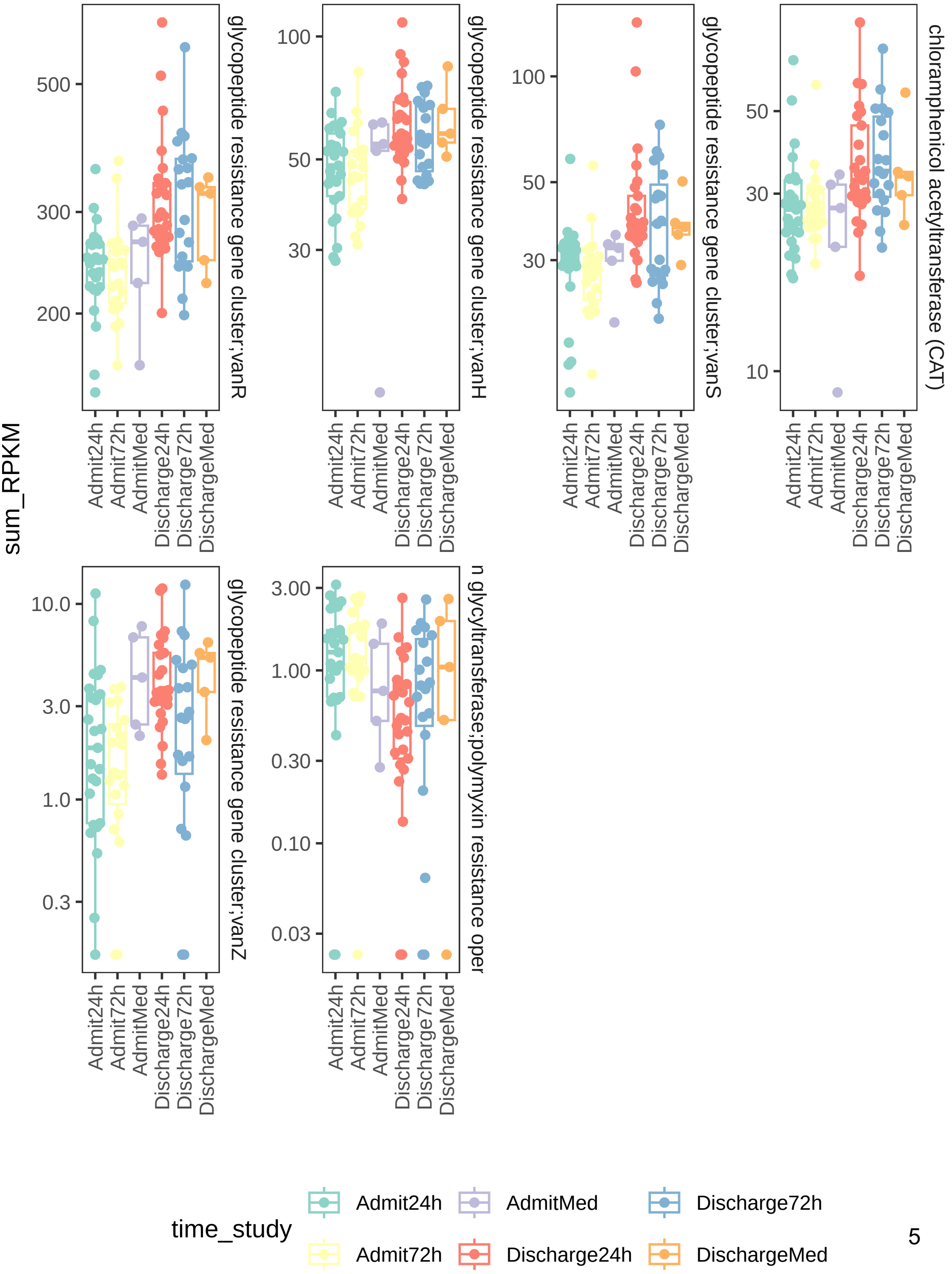
sum_RPKM
glycopeptide resistance gene cluster;vanR
glycopeptide resistance gene cluster;vanH
glycopeptide resistance gene cluster;vanS
chloramphenicol acetyltransferase (CAT)
glycopeptide resistance gene cluster;vanZ
n glycyltransferase;polymyxin resistance oper
500
300
200
100
50
30
10
10.0
3.0
1.0
0.3
3.00
1.00
0.30
0.10
0.03
Admit24h
Admit72h
AdmitMed
Discharge24h
Discharge72h
DischargeMed
time_study

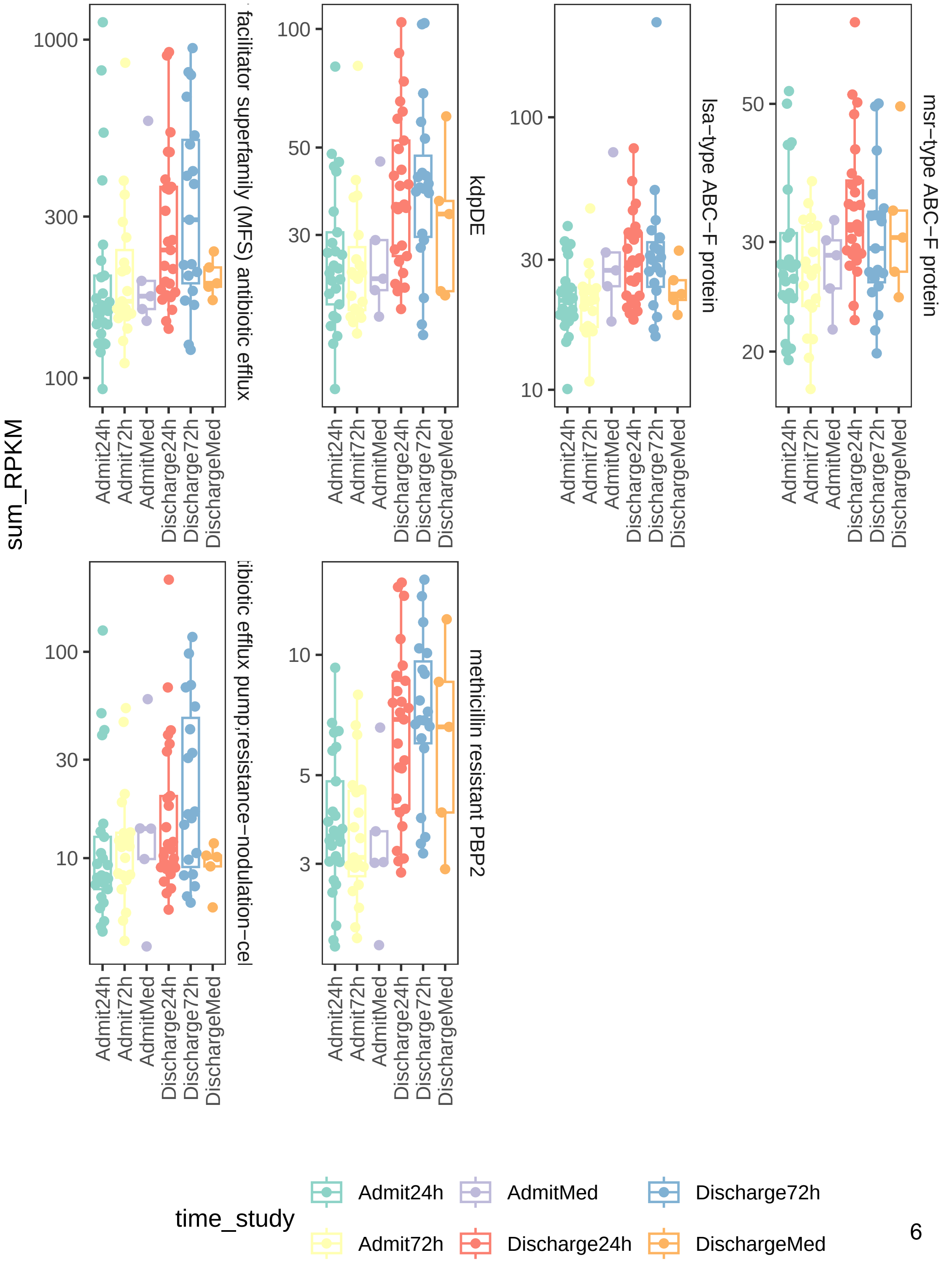

sum_RPKM
facilitator superfamily (MFS) antibiotic efflux
kdpDE
lsa−type ABC−F protein
msr−type ABC−F protein
ibiotic efflux pump;resistance−nodulation−cel
methicillin resistant PBP2
Admit24h
Admit72h
AdmitMed
Discharge24h
Discharge72h
DischargeMed
time_study

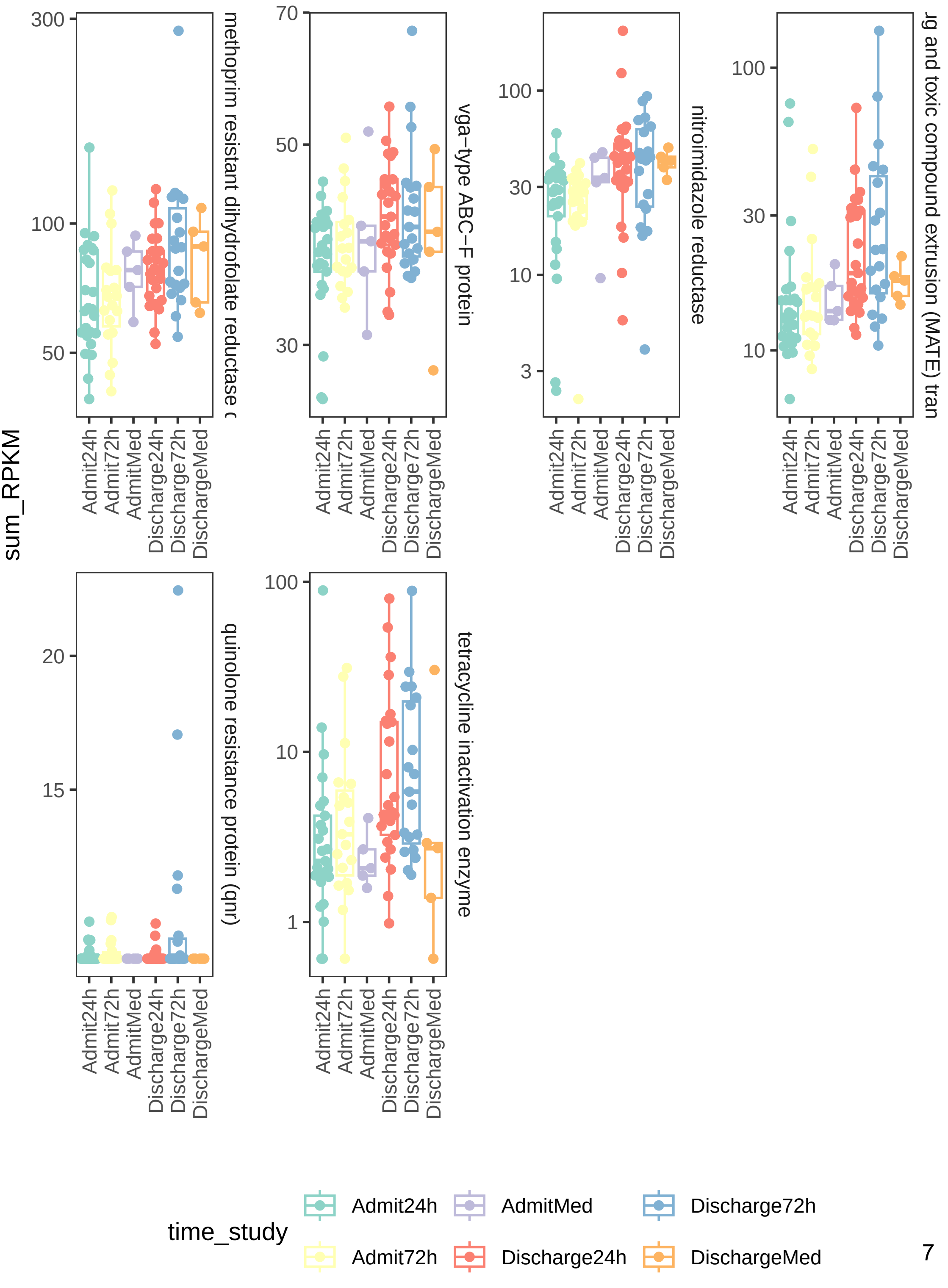
sum_RPKM
methoprim resistant dihydrofolate reductase c
vga−type ABC−F protein
nitroimidazole reductase
ug and toxic compound extrusion (MATE) tran
quinolone resistance protein (qnr)
tetracycline inactivation enzyme
Admit24h
Admit72h
AdmitMed
Discharge24h
Discharge72h
DischargeMed
time_study

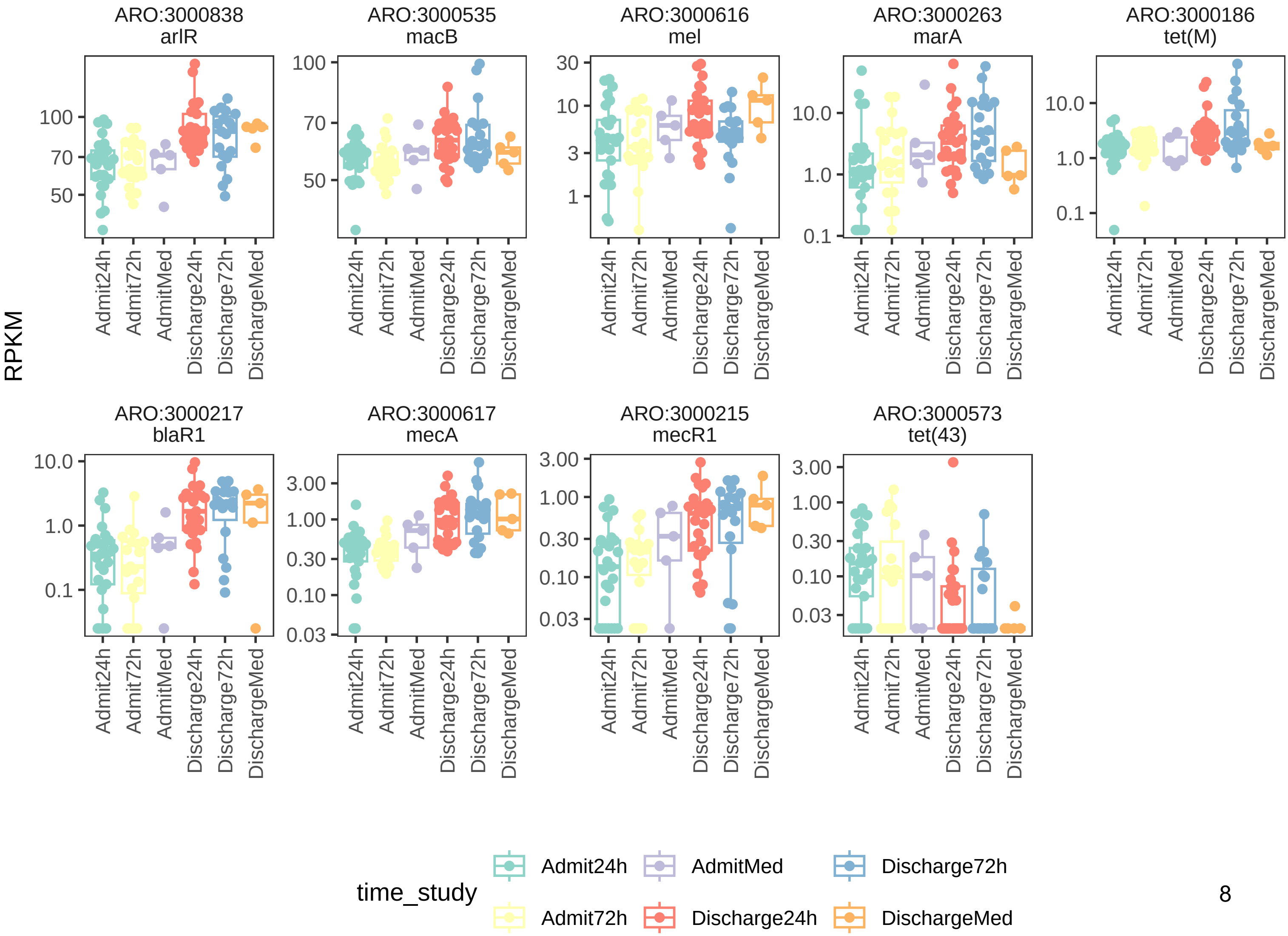
ARO:3000838
arlR
ARO:3000535
macB
ARO:3000616
mel
ARO:3000263
marA
ARO:3000186
tet(M)
ARO:3000217
blaR1
ARO:3000617
mecA
ARO:3000215
mecR1
ARO:3000573
tet(43)
RPKM
Admit24h
Admit72h
AdmitMed
Discharge24h
Discharge72h
DischargeMed
time_study

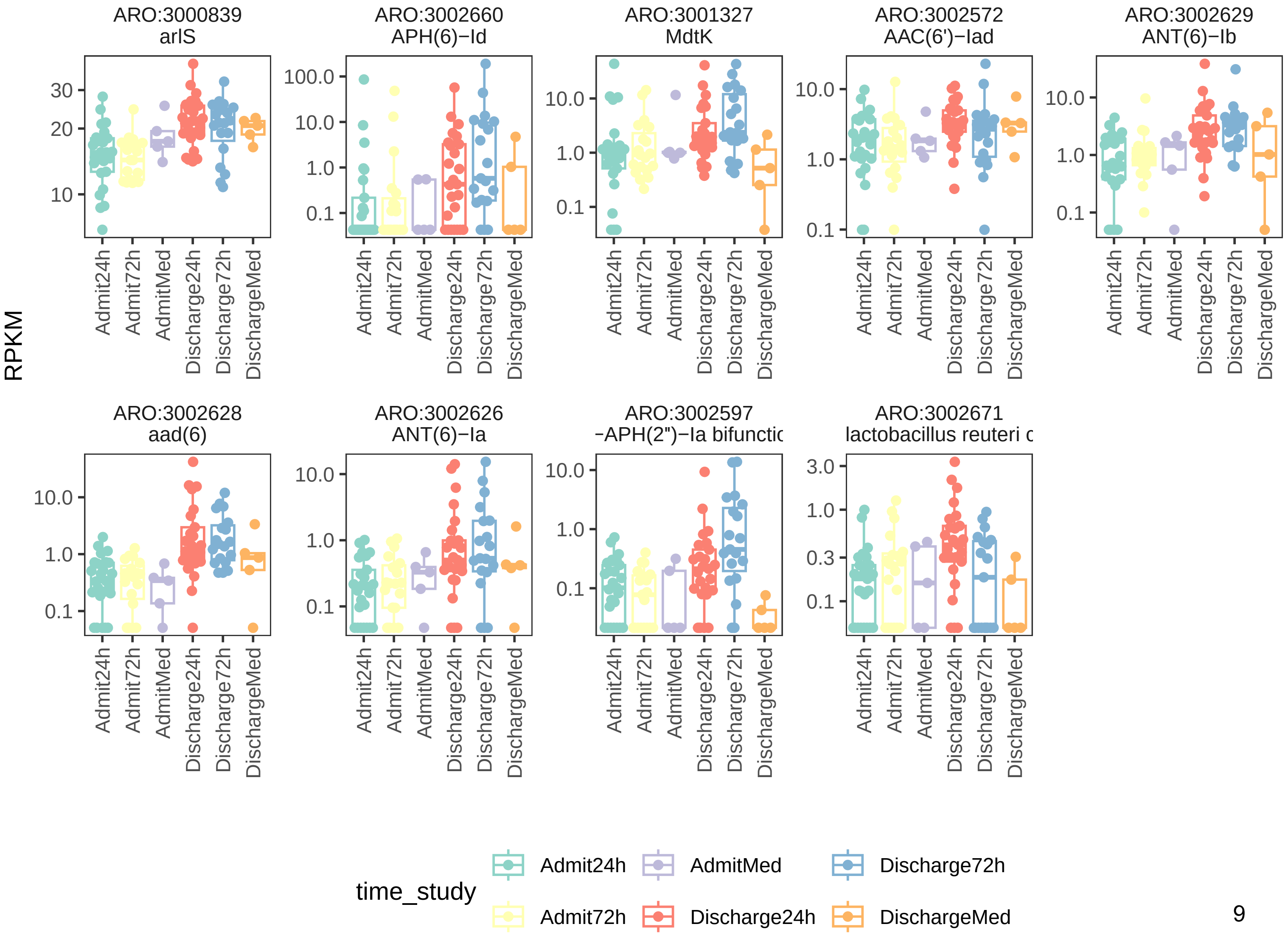
ARO:3000839
arlS
ARO:3002660
APH(6)−Id
ARO:3001327
MdtK
ARO:3002572
AAC(6')−Iad
ARO:3002629
ANT(6)−Ib
ARO:3002628
aad(6)
ARO:3002626
ANT(6)−Ia
ARO:3002597
−APH(2")−Ia bifunctic
ARO:3002671
lactobacillus reuteri c
RPKM
Admit24h
Admit72h
AdmitMed
Discharge24h
Discharge72h
DischargeMed
time_study

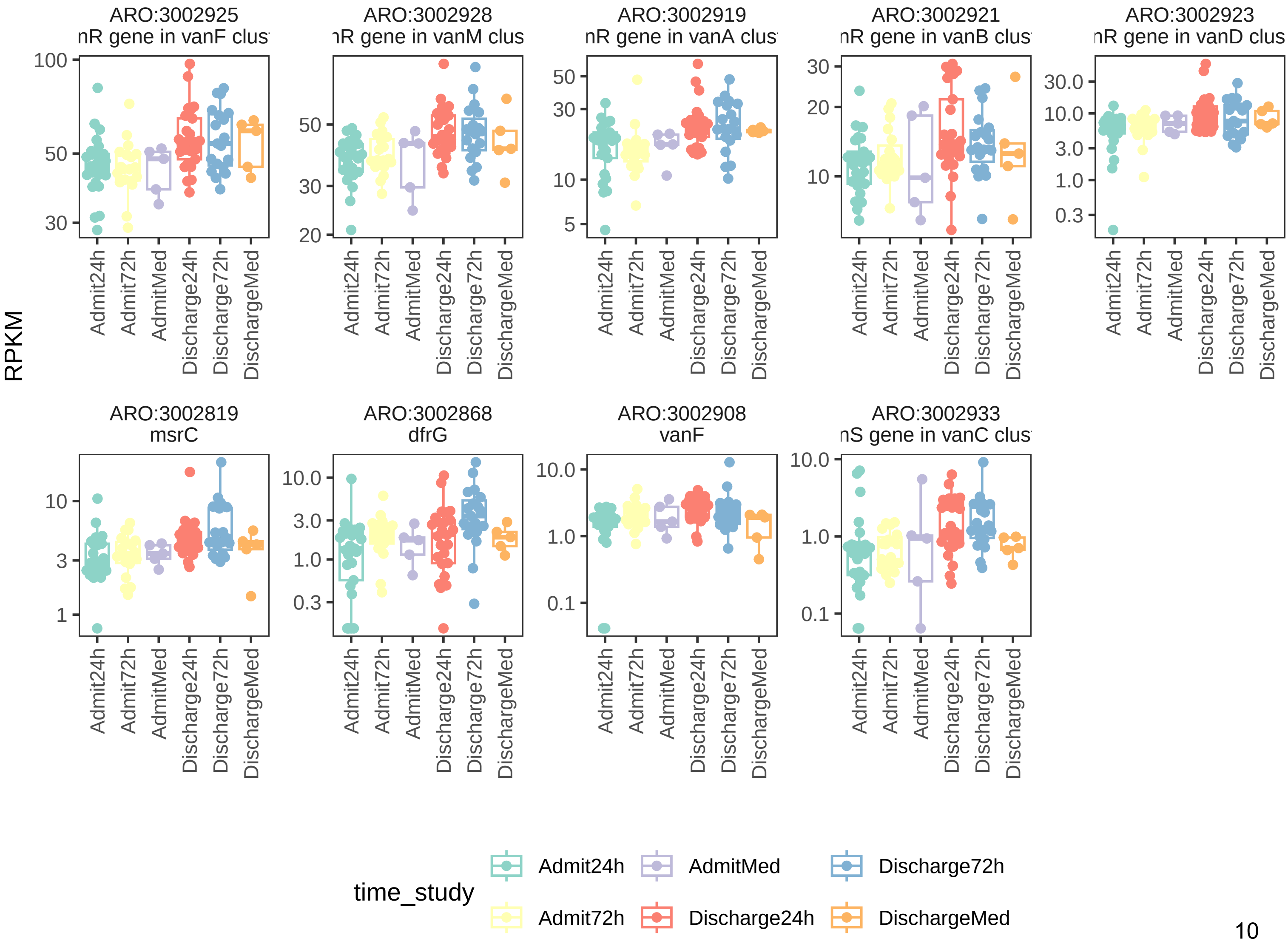
ARO:3002925
nR gene in vanF clus
ARO:3002928
nR gene in vanM clus
ARO:3002919
nR gene in vanA clus
ARO:3002921
nR gene in vanB clus
ARO:3002923
nR gene in vanD clus
ARO:3002819
msrC
ARO:3002868
dfrG
ARO:3002908
vanF
ARO:3002933
nS gene in vanC clus
RPKM
Admit24h
Admit72h
AdmitMed
Discharge24h
Discharge72h
DischargeMed
time_study

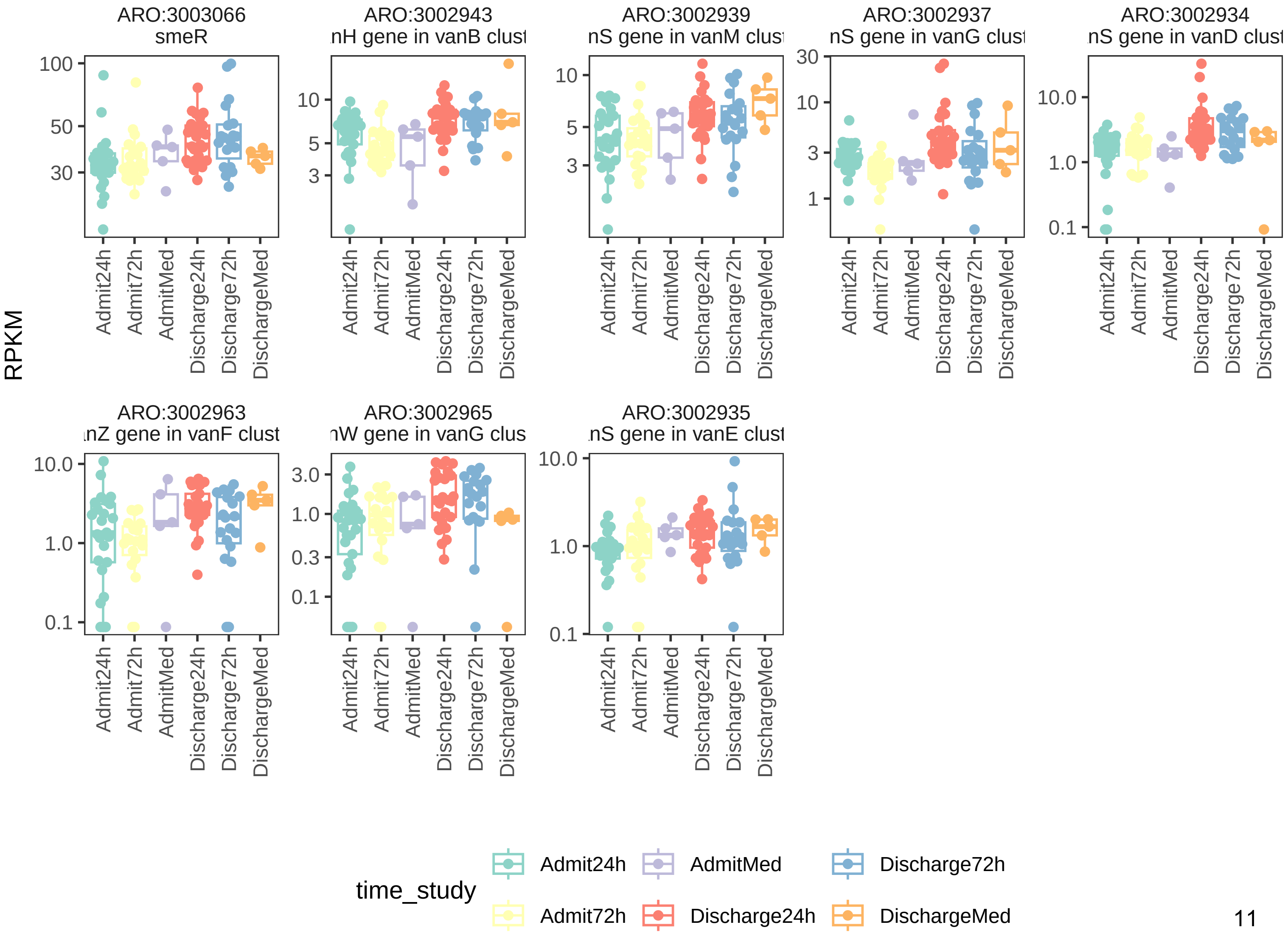
ARO:3003066
smeR
ARO:3002943
nH gene in vanB clust
ARO:3002939
nS gene in vanM clust
ARO:3002937
nS gene in vanG clust
ARO:3002934
nS gene in vanD clust
ARO:3002963
nZ gene in vanF clust
ARO:3002965
nW gene in vanG clus
ARO:3002935
nS gene in vanE clust
RPKM
Admit24h
Admit72h
AdmitMed
Discharge24h
Discharge72h
DischargeMed
time_study

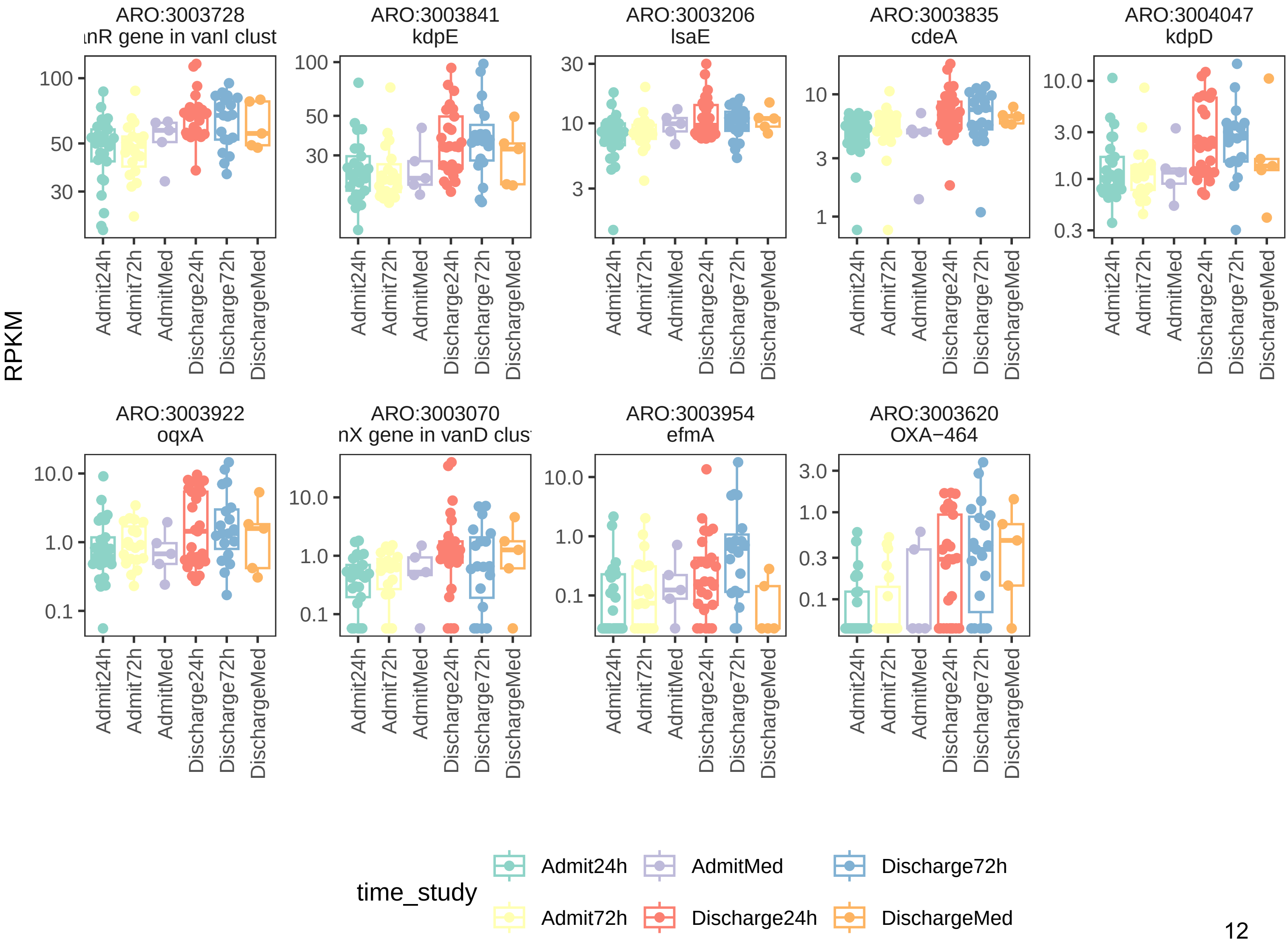

ARO:3003728
ınR gene in vanI clust
ARO:3003841
kdpE
ARO:3003206
lsaE
ARO:3003835
cdeA
ARO:3004047
kdpD
ARO:3003922
oqxA
ARO:3003070
nX gene in vanD clus
ARO:3003954
efmA
ARO:3003620
OXA−464
RPKM
Admit24h
Admit72h
AdmitMed
Discharge24h
Discharge72h
DischargeMed
time_study

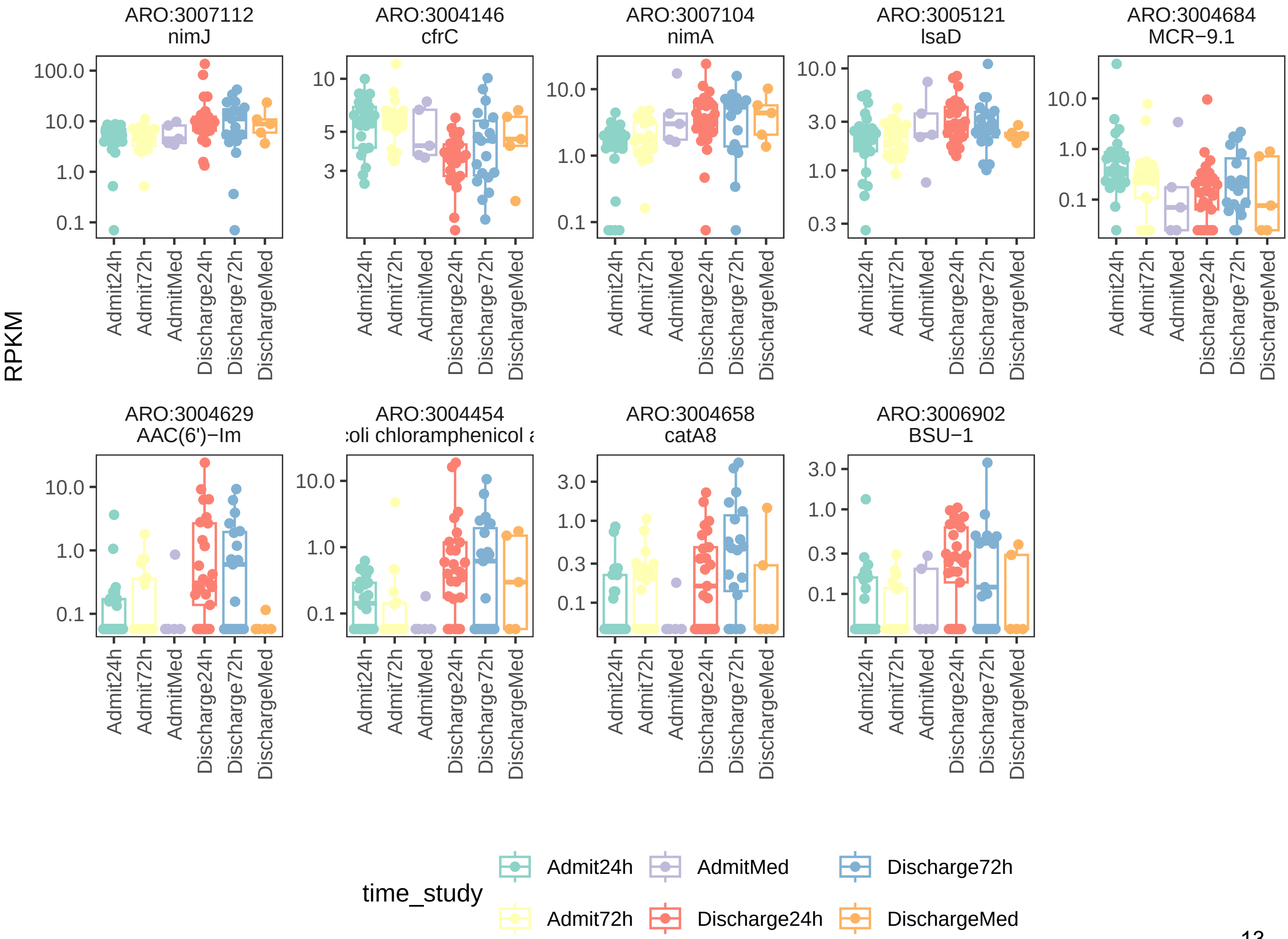
ARO:3007112
nimJ
ARO:3004146
cfrC
ARO:3007104
nimA
ARO:3005121
lsaD
ARO:3004684
MCR−9.1
ARO:3004629
AAC(6')−Im
ARO:3004454
:oli chloramphenicol a
ARO:3004658
catA8
ARO:3006902
BSU−1
RPKM
Admit24h
Admit72h
AdmitMed
Discharge24h
Discharge72h
DischargeMed
time_study
