# Supplementary material for: Effect of surgical antimicrobial prophylaxis duration for colic surgery on complications and resistome
Source: Equine Vet J. 2025 Dec 10;58(2):390–403. doi: 10.1002/evj.70137 (PMC12892381; doi:10.1002/evj.70137)
Supplement: Supplementary file 7 — Figure S6. Abundance of TEM beta‐lactamase genes at discharge in the 24 h group vs. the 72 h group. Panel A shows a heat map of three specific TEM beta‐lactamase genes (TEM‐117, TEM‐126, and TEM‐192) across patient samples at admission and discharge in the 24 h and 72 h group. Colour intensity reflects gene abundance after adding a small pseudocount to handle undetected genes (i.e., zero values), with higher expression shown in dark blue and lower expression in yellow/white. Rows represent genes, and columns represent individual patient samples, grouped by timepoint and group. Panel B shows the results of a zero‐inflated beta regression model that accounts for the large number of samples where specific genes were not detected. This model estimates the probability that each gene was increased or decreased relative to its level in the 24 h group. Points to the right of the dashed line indicate increased abundance in the 72 h group compared to the 24 h group. Filled circles denote statistically significant changes. TEM‐126 and TEM‐117 were significantly more abundant in the 72 h group, while TEM‐192 was not significantly different. [file EVJ-58-390-s002.pdf]

Equine Veterinary Journal BEVA

Figure S6: Abundance of TEM beta-lactamase genes at discharge in the 24h group vs. the 72h group.

Panel A shows a heat map of three specific TEM beta-lactamase genes (TEM-117, TEM-126, and TEM-192) across patient samples at admission and discharge in the 24h and 72h group. Color intensity reflects gene abundance after adding a small pseudocount to handle undetected genes (i.e., zero values), with higher expression shown in dark blue and lower expression in yellow/white. Rows represent genes, and columns represent individual patient samples, grouped by timepoint and group.

Panel B shows the results of a zero-inflated beta regression model that accounts for the large number of samples where specific genes were not detected. This model estimates the probability that each gene was increased or decreased relative to its level in the 24h group. Points to the right of the dashed line indicate increased abundance in the 72 hr group compared to the 24 hr group. Filled circles denote statistically significant changes. TEM-126 and TEM-117 were significantly more abundant in the 72h group, while TEM-192 was not significantly different.

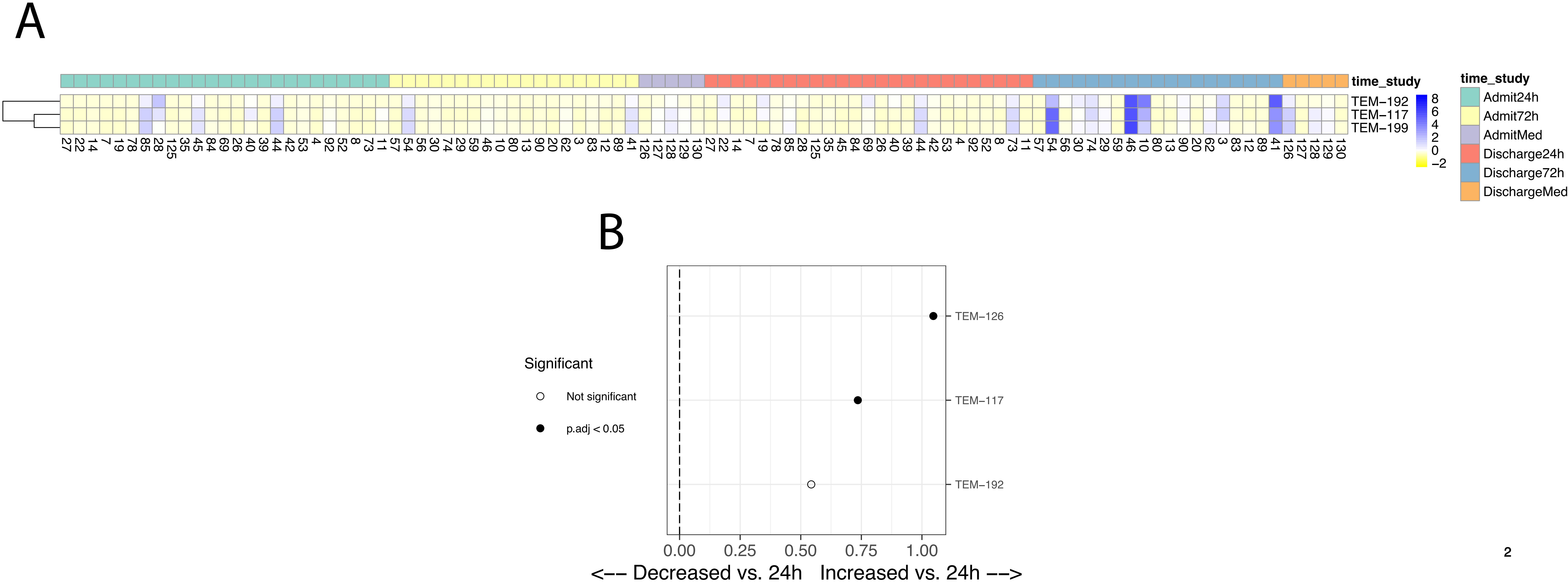

A
time_study
TEM−192
TEM−117
TEM−199
time_study
Admit24h
Admit72h
AdmitMed
Discharge24h
Discharge72h
DischargeMed
B
Significant
Not significant
p.adj < 0.05
TEM−126
TEM−117
TEM−192
0.00
0.25
0.50
0.75
1.00
<−− Decreased vs. 24h Increased vs. 24h −−>
