# Supplementary material for: Effect of surgical antimicrobial prophylaxis duration for colic surgery on complications and resistome
Source: Equine Vet J. 2025 Dec 10;58(2):390–403. doi: 10.1002/evj.70137 (PMC12892381; doi:10.1002/evj.70137)
Supplement: Supplementary file 8 — Table S1. Specific reasons for exclusion for each horse. [file EVJ-58-390-s005.pdf]

Table S1: Specific reasons for exclusion for each horse.

Envelope Number	AMD group	Reason for exclusion
1	72	Developed azotemia postoperatively (creatinine concentration 2.25 mg/dL) so only received one dose of gentamicin. Mild episode colic 70 hours postoperatively that resolved without treatment. Abdominocentesis performed 72 hours postoperatively due to more persistent colic signs; peritoneal fluid serosanguinous with a lactate concentration of 1.3 mmol/L, total solids of 4.4 g/dL, and nucleated cell count not performed. Euthanased 4 days postoperatively for post-operative colic and no option for re-lap (for financial and emotional reasons)
17	24	Euthanased 72 hours after surgery for strangulating lipoma affecting the distal ileum. No resection. Euthanased because of persistent tachycardia, recumbency, and postoperative reflux. No SSI.
26	72	Large colon volvulus euthanased 4 days after surgery because of presumed colonic infarction.
33	24	Diagnosed with proximal enteritis and mistakenly given an additional dose of gentamicin.
34	72	Large colon volvulus euthanased 24 hours after surgery because of shock.
37	24	Euthanased 4 days after surgery for a strangulating pedunculated lipoma and jejunojejunostomy because of persistent postoperative ileus. No SSI.
49	72	Euthanased 72 hours after surgery for a caecal impaction because of caecal rupture.
53	72	Removed from the study because of a newly diagnosed azotemia.
61	72	Euthanased 24 hours after surgery because of a diaphragmatic hernia not diagnosed during surgery.
69	72	Euthanased 24 hours after surgery for a small intestinal strangulation caused by a GIST because of persistent colic of undetermined cause.
72	24	Large colon volvulus euthanased 4 days after surgery because of colonic infarct and multiorgan venous thrombosis.
82	24	Excluded because antimicrobial drugs were re-started 2 days postoperatively due to severe aspiration pneumonia that occurred during general anaesthesia. Horse was ultimately euthanased due to laminitis. No SSI.
83	24	Large colon volvulus euthanased 6 hours after surgery because of shock.
99	72	Euthanased 72 hours after surgery for volvulus nodosus and jejunoileostomy. At relaparotomy there was separation of her body wall at the previous surgical site, and compromise to the jejunum orad to the previous anastomosis which would have necessitated resection and jejunoceacostomy.

106	24	Large colon volvulus euthanased 48 hours after surgery because of shock.
108	24	Aborted pregnancy 72 hours after surgery for a small intestinal strangulation through a dorsal body wall rent. Retained fetal membranes requiring re-starting antimicrobials.
131	72	Euthanased within 24 hours postoperatively because of persistent colic caused by a segmental jejunal infarct.
137	72	Euthanased 9 days after surgery for strangulating pedunculated lipoma of the descending colon (no resection performed). Repeat celiotomy resection and anastomosis of the small colon performed. Reason for euthanasia septic peritonitis caused by focal transmural necrosis of the descending colon.
140	24	Euthanased 9 days after surgery for a caecal impaction because of persistent colic signs. Possible pneumonia; no SSI
152	72	Septic thrombophlebitis preoperatively (reason for exclusion). Sand and gravel enteropathy/impaction. Euthanased day 7 for colic and septic peritonitis.
