# Supplementary material for: Effect of surgical antimicrobial prophylaxis duration for colic surgery on complications and resistome
Source: Equine Vet J. 2025 Dec 10;58(2):390–403. doi: 10.1002/evj.70137 (PMC12892381; doi:10.1002/evj.70137)
Supplement: Supplementary file 9 — Table S2. Signalment, physical exam, and clinicopathologic variables for horses enroled in a clinical trial comparing 24‐ and 72‐h of surgical antimicrobial prophylaxis for colic surgery. [file EVJ-58-390-s009.pdf]

Table S2: Signalment, physical exam, and clinicopathologic variables for horses enrolled in a clinical trial comparing 24- and 72-hours of surgical antimicrobial prophylaxis for colic surgery.

**Variable**	**24-hour group**	**72-hour group**	**P-value**
Median (IQR) age (years)[N]	16 (9, 20) [71]	12 (6,18) [69]	0.03
Median (IQR) bodyweight (kg)[N]	533 (470-590) [71]	530 (488, 581) [68]	0.9
Breed – N (%)			0.7
American	24 (34%)	20 (29%)	
Thoroughbred	16 (23%)	20 (29%)	
Warmblood	15 (21%)	11 (16%)	
Standardbred	10 (14%)	10 (14%)	
Other/Cross	5 (7%)	4 (6%)	
Arabian	1 (1%)	4 (6%)	
Nasogastric reflux – N (%)	8 (11%)	10 (14%)	0.6
Median (IQR) nasogastric reflux volume (L) [N]	0 (0, 0) [71]	0 (0, 0.25) [66]	0.3
Median (IQR) packed cell volume (L/L) [N]	0.40 (0.34, 0.44) [71]	0.39 (0.35, 0.44) [69]	0.9
Median (IQR) total solids (g/L) [N]	68 (62, 74) [71]	70 (64, 76) [69]	0.3
Median (IQR) blood lactate concentration (mmol/L)[N]	1.6 (0.9, 2.9) [71]	1.6 (0.9, 2.7) [69]	0.6
Median (IQR) blood glucose concentration (mmol/L) [N]	9.7 (7.1, 11.9) [70]	9.3 (7.2, 11.6) [69]	0.4

Median (IQR) plasma creatinine concentration (mmol/L) [N]	123 (103, 139) [69]	114 (99, 138) [68]	0.3
Median (IQR) plasma ionised calcium concentration (mmol/L)[N]	1.37 (1.30, 1.45) [69]	1.41 (1.32, 1.48) [69]	0.4
Median (IQR) plasma sodium (mEq/L)[N]	135 (133, 137) [70]	136 (134, 138) [69]	0.07
Median (IQR) plasma chloride (mEq/L)[N]	97 (93, 100) [68]	98 (96, 101) [69]	0.08
Median (IQR) plasma potassium (mEq/L)[N]	3.5 (3.2, 3.7) [70]	3.4 (3.2, 3.7) [69]	0.7
Median (IQR) peritoneal fluid TS (g/L)[N]	30 (20, 38) [32]	25 (19, 36) [25]	0.8
Median (IQR) peritoneal fluid lactate (mmol/L)[N]	3.7 (2.6, 5.6) [35]	4.6 (2.8, 6.2) [33]	0.3
Peritoneal nucleated cell count (x10^9 cells/L)[N]	1.44 (1.36, 3.30) [3]	29.55 (0.76, 60.31) [4]	>0.9

IQR, interquartile range shown as 25th and 75th percentile; TS, total solids, [N], number of cases for which the information was available.
