# Supplementary material for: Effect of surgical antimicrobial prophylaxis duration for colic surgery on complications and resistome
Source: Equine Vet J. 2025 Dec 10;58(2):390–403. doi: 10.1002/evj.70137 (PMC12892381; doi:10.1002/evj.70137)
Supplement: Supplementary file 10 — Table S3. Surgical/general anaesthesia and post‐operative treatment variables for horses enroled in a clinical trial comparing 24‐ and 72‐h of surgical antimicrobial prophylaxis for colic surgery. [file EVJ-58-390-s006.pdf]

Table S3: Surgical/general anaesthesia and post-operative treatment variables for horses enrolled in a clinical trial comparing 24- and 72-hours of surgical antimicrobial prophylaxis for colic surgery.

Variable	24-hour group	72-hour group	P-value
Time between administration of first perioperative antimicrobial dose and start of surgery (min) [N]	15 (10, 29) [70]	15 (10, 25) [69]	0.8
Median (IQR) duration of anaesthesia (min) [N]	185 (135, 225) [71]	155 (130, 213) [69]	0.2
Median (IQR) duration of surgery (min) [N]	145 (95, 200) [71]	120 (90, 175) [69]	0.2
Median (IQR) lowest recorded MAP (mmHg) [N]	65 (62, 70) [71]	68 (64, 72) [69]	0.3
Median (IQR) lowest recorded PaO_2 (mmHg) [N]	191 (90, 340) [71]	143 (94, 295) [69]	0.2
Median (IQR) highest recorded $PaCO_2$ (mmHg) [N]	53 (50, 58) [71]	54 (48, 59) [69]	0.7
Median (ICR) intraoperative PCV (L/L) [N]	0.40 (0.36, 0.45) [62]	0.41 (0.38, 0.45) [57]	0.8
Median (IQR) intraoperative TS (g/L) [N]	52 (48, 58) [59]	54 (50, 58) [54]	0.4
Median (IQR) recovery duration (min) [N]	55 (39, 74) [71]	55 (40, 70) [69]	1.0
Lesion location – N (%)			0.8
Small intestine (SI)	40 (56%)	37 (54%)	
Large intestine (LI)	30 (42%)	30 (43%)	
SI/LI	1 (2%)	2 (3%)	

Lesion type – N (%)			0.3
Strangulating	52 (73%)	45 (65%)	
Non-strangulating	19 (27%)	24 (35%)	
Procedure category - N (%)			0.4
Exploratory laparotomy/reposition	31 (44%)	33 (48%)	
Small intestinal resection/anastomosis	21 (30%)	20 (29%)	
Pelvic flexure enterotomy +/- enema	10 (14%)	13 (19%)	
Other LI enterotomy/enterectomy	5 (7%)	1 (1%)	
Re-dosed with potassium penicillin – N (%)			0.8
Re-dosed once	30 (42%)	31 (45%)	
Re-dosed twice	3 (4%)	4 (6%)	
Not re-dosed	38 (54%)	34 (49%)	
Wound protection during recovery – N (%)			0.9
Iodine-impregnated adhesive drape (Ioban®)	55 (79%)	55 (80%)	
Stent bandage with an Ioban®	10 (14%)	10 (14%)	
Stent bandage only	5 (7%)	4 (6%)	
Surgeon – N (%)			0.9
Surgeon	46 (65%)	42 (61%)	
Junior	12 (18%)	13 (19%)	

Resident	13 (17%)	14 (20%)	
Wound protection *not* dislodged – N (%)	66 (94%)	66 (96%)	1.0
Post-recovery abdominal bandage applied – N (%)	35 (50%)	26 (38%)	0.1
Median (IQR) duration of treatment with flunixin meglumine [N]	104 (84, 120) [71]	108 (86, 120) [69]	0.7
Lidocaine CRI administration – N (%)	62 (87%)	55 (80%)	0.3
Median (IQR) duration of lidocaine CRI (h)[N]	24 (20, 40) [62]	30 (18, 48) [55]	
Median (IQR) fluid rate (L/h) [N]	1.5 (1, 2) [71]	1.5 (1, 2) [69]	0.7
Median (IQR) duration of fluid therapy (h) [N]	24 (20, 40) [71]	30 (20, 48) [69]	0.6
IV fluid supplementation – N (%)			0.5
KCl	32 (45%)	29 (42%)	
KCl/calcium gluconate	10 (14%)	10 (14%)	
KCl/calcium gluconate/dextrose	5 (7%)	4 (6%)	
Other†	11 (16%)	6 (9%)	
No supplementation	13 (18%)	20 (29%)	
Median (IQR) time to first feed (h)[N]	20 (12, 24) [71]	24 (12, 33)[69]	0.2
Type of first feed – N (%)			0.8
Equine senior pellets	47 (66%)	40 (58%)	
Alfalfa hay	16 (23%)	16 (23%)	
Other	8 (11%)	13 (19%)	

Median (IQR) time to full feed (h) [N]	120 (84, 144) [68]	120 (84, 167) [68]	0.7
Type of full feed – N (%)			0.7
Free-choice mixed hay<br>Equine senior pellets	25 (35%)	20 (29%)	
Free-choice mixed hay only	18 (25%)	22 (32%)	
Timothy hay<br>Equine senior pellets	8 (11%)	11 (16%)	
Timothy hay only	5 (7%)	5 (7%)	
Alfalfa hay<br>Equine senior pellets	2 (3%)	5 (7%)	
Alfalfa hay only	4 (7%)	3 (5%)	
Other	6 (8%)	2 (3%)	
Not on full feed at discharge or euthanasia	3 (4%)	1 (1%)	

Abbreviations see Table S2. MAP, mean arterial pressure; PCV, packed cell volume; TS, total solids; CRI, constant rate infusion; †combination of KCl, calcium gluconate, dextrose, magnesium or thiamine
