# Supplementary material for: Effect of surgical antimicrobial prophylaxis duration for colic surgery on complications and resistome
Source: Equine Vet J. 2025 Dec 10;58(2):390–403. doi: 10.1002/evj.70137 (PMC12892381; doi:10.1002/evj.70137)
Supplement: Supplementary file 11 — Table S4. Bacteria isolated on culture from the incisional infection. [file EVJ-58-390-s004.pdf]

Equine Veterinary Journal BEVA

Table S4: Bacteria isolated on culture from the incisional infection.

Surgical antimicrobial prophylaxis group	Organisms identified on culture	Number of horses
24-hour	*Streptococcus equi* subsp zooepidemicus	1
	Enterobacter cloacae complex/ *Enterococcus faecium* (2 listed are MDR)/*S. equi* subsp zooepidemicus	1
	Escherichia coli/*Klebsiella pneumoniae*/*E. cloacae* complex (3 listed are MDR)/*S. aureus*	1
	E. coli	1
	Staphylococcus aureus and light mixed of other organisms	1
	No growth	1
	No sample	6[*#]
72-hour	*Citrobacter freundii* complex	1
	K. pneumonia/*E. coli*/*E. cloacae* complex/E. *faecium (all listed are MDR)*	1
	E. coli/*E. cloacae* complex/*Acinetobacter baumannii*/*Pseudomonas aeruginosa*/*Bacteroides fragilis* (all listed are MDR)	1
	Staphylococcus epidermis/ *Aeromonas hydrophila* (all listed are MDR)	1
	S. aureus (MDR)	1

	No growth	1
	No sample	5*

MDR, multi-drug-resistant organism(s). * Horses had drainage that was mild and/or observed post-discharge; # one horse had septic peritonitis that grew multiple enteric organisms, and the surgical site infection was thought to be associated with the peritonitis.
