# Supplementary material for: Effect of surgical antimicrobial prophylaxis duration for colic surgery on complications and resistome
Source: Equine Vet J. 2025 Dec 10;58(2):390–403. doi: 10.1002/evj.70137 (PMC12892381; doi:10.1002/evj.70137)
Supplement: Supplementary file 12 — Table S5. Percentage of host‐derived sequencing reads removed during quality control processing. This table shows the proportion of raw sequencing reads that were identified as host DNA (e.g., horse) and removed during quality control filtering prior to downstream metagenomic analysis. Removing host reads is a standard step to ensure analysis focuses on microbial DNA. [file EVJ-58-390-s011.pdf]

Equine Veterinary Journal BEVA

Table S5: Percentage of host-derived sequencing reads removed during quality control processing.

HorseID	timepoint	study_group	Host reads	Non-host reads	Percent host reads
45	Admit	24h	11324161	1450307	88.65
54	Discharge	72h	1308458	350859	78.86
40	Admit	24h	9589064	6508946	59.57
128	Admit	Med	2256174	2180548	50.85
53	Admit	24h	2189445	5543614	28.31
46	Discharge	72h	68435	303020	18.42
53	Discharge	24h	1528510	8987038	14.54
62	Admit	72h	690522	4248151	13.98
19	Admit	24h	1637385	11292537	12.66
73	Discharge	24h	155985	1166798	11.79
52	Admit	24h	1114295	8431706	11.67
54	Admit	72h	141683	1146866	11
69	Discharge	24h	478936	4081018	10.5
10	Admit	72h	579392	5148074	10.12
22	Admit	24h	1024238	9284771	9.94
11	Admit	24h	735596	6840104	9.71
129	Admit	Med	364936	3532287	9.36
22	Discharge	24h	490336	5167145	8.67
44	Discharge	24h	107778	1192853	8.29
89	Admit	72h	918666	10768570	7.86
127	Admit	Med	299481	3524385	7.83
46	Admit	72h	345370	4378057	7.31
39	Admit	24h	1105019	14232191	7.2
12	Admit	72h	935025	12141467	7.15
3	Admit	72h	880056	11672100	7.01
14	Admit	24h	623308	8525780	6.81
12	Discharge	72h	726891	10373296	6.55
27	Admit	24h	789417	11303962	6.53
28	Admit	24h	537583	7774442	6.47
126	Admit	Med	609552	8836893	6.45
59	Admit	72h	842648	12224458	6.45
89	Discharge	72h	485347	7042965	6.45
128	Discharge	Med	296180	4315773	6.42
129	Discharge	Med	190486	2777290	6.42
90	Admit	72h	506592	7404185	6.4
92	Discharge	24h	573424	8408079	6.38
130	Admit	Med	418247	6302613	6.22
4	Discharge	24h	807093	12176550	6.22
56	Admit	72h	539932	8151064	6.21
8	Admit	24h	776683	11922325	6.12
125	Admit	24h	696409	10769448	6.07
57	Admit	72h	704948	10981077	6.03
7	Admit	24h	896681	14011066	6.01
41	Admit	72h	93223	1462218	5.99
125	Discharge	24h	476830	7544953	5.94
74	Admit	72h	658452	10525328	5.89
30	Admit	72h	322352	5202990	5.83

14	Discharge	24h	589668	9693216	5.73
4	Admit	24h	665332	10974003	5.72
126	Discharge	Med	56375	932014	5.7
26	Admit	24h	825373	13813018	5.64
59	Discharge	72h	784562	13332692	5.56
80	Admit	72h	384855	6542222	5.56
90	Discharge	72h	445906	7668048	5.5
42	Admit	24h	410244	7080601	5.48
52	Discharge	24h	403934	7076651	5.4
20	Admit	72h	863765	15153132	5.39
13	Discharge	72h	570394	10024464	5.38
20	Discharge	72h	542354	9556132	5.37
30	Discharge	72h	165306	2973665	5.27
41	Discharge	72h	75404	1415331	5.06
7	Discharge	24h	462749	8978804	4.9
39	Discharge	24h	477784	9363276	4.86
35	Discharge	24h	464996	9129052	4.85
13	Admit	72h	522968	10332032	4.82
85	Admit	24h	48426	957688	4.81
127	Discharge	Med	796445	16258218	4.67
62	Discharge	72h	123477	2554849	4.61
3	Discharge	72h	266811	5569003	4.57
83	Discharge	72h	337360	7089176	4.54
44	Admit	24h	44964	948680	4.53
74	Discharge	72h	580490	12475620	4.45
69	Admit	24h	496200	10736622	4.42
8	Discharge	24h	424096	9264719	4.38
57	Discharge	72h	595379	13059501	4.36
92	Admit	24h	165874	3648857	4.35
84	Discharge	24h	365742	8757443	4.01
29	Admit	72h	364547	8973723	3.9
73	Admit	24h	472252	11759362	3.86
42	Discharge	24h	202770	5327739	3.67
78	Discharge	24h	199771	5259635	3.66
80	Discharge	72h	469956	12456712	3.64
35	Admit	24h	291695	7978239	3.53
11	Discharge	24h	392316	10801329	3.5
27	Discharge	24h	318410	8877227	3.46
28	Discharge	24h	401750	11226070	3.46
10	Discharge	72h	318241	9501955	3.24
78	Admit	24h	235107	7262120	3.14
84	Admit	24h	216189	6695475	3.13
130	Discharge	Med	289652	9168646	3.06
85	Discharge	24h	98123	3258069	2.92
26	Discharge	24h	411821	13726855	2.91
45	Discharge	24h	392821	13658871	2.8
29	Discharge	72h	76826	2933075	2.55
40	Discharge	24h	317672	12614389	2.46
19	Discharge	24h	322273	13561128	2.32
56	Discharge	72h	280257	12023961	2.28
83	Admit	72h	116368	5099776	2.23

This table shows the proportion of raw sequencing reads that were identified as host DNA (e.g., horse) and removed during quality control filtering prior to downstream metagenomic analysis. Removing host reads is a standard step to ensure analysis focuses on microbial DNA.
