# Supplementary material for: Effect of surgical antimicrobial prophylaxis duration for colic surgery on complications and resistome
Source: Equine Vet J. 2025 Dec 10;58(2):390–403. doi: 10.1002/evj.70137 (PMC12892381; doi:10.1002/evj.70137)
Supplement: Supplementary file 2 — Figure S1. Heatmap of bacterial taxa across time points and groups, including positive and negative control samples. [file EVJ-58-390-s001.pdf]

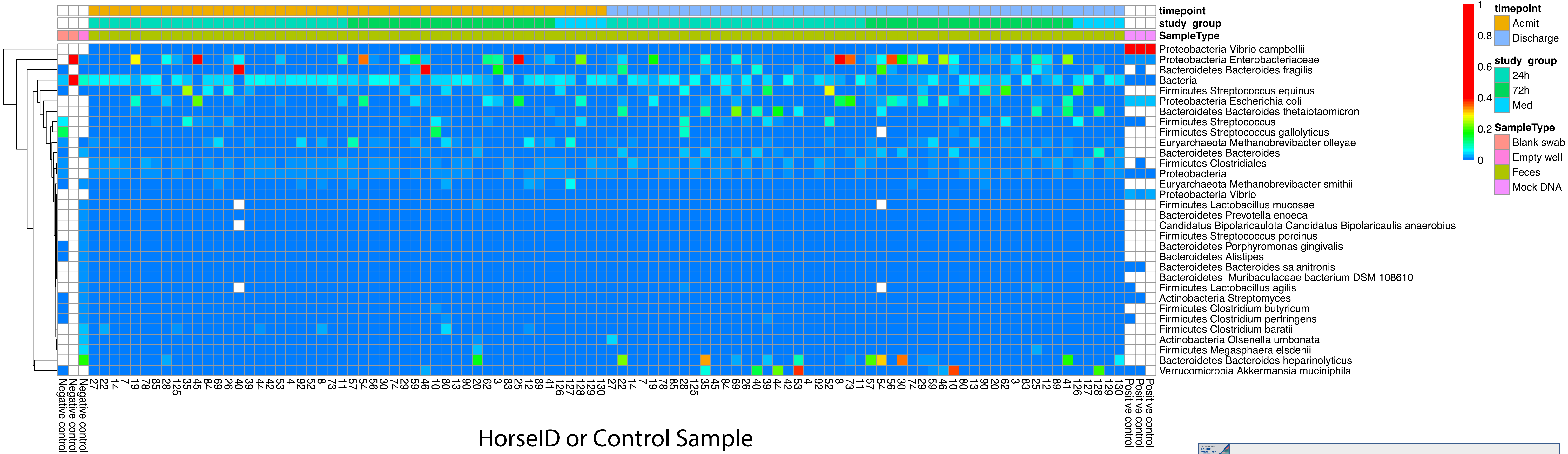

Equine Veterinary Journal BEVA

Figure S1: Heatmap of bacterial taxa across time points and groups, including positive and negative control samples.
